# Novel modulators of p53-signaling encoded by unknown genes of emerging viruses

**DOI:** 10.1371/journal.ppat.1009033

**Published:** 2021-01-07

**Authors:** Dina Alzhanova, Kathleen Corcoran, Aubrey G. Bailey, Kristin Long, Sharon Taft-Benz, Rachel L. Graham, Grant S. Broussard, Mark Heise, Gabriele Neumann, Peter Halfmann, Yoshihiro Kawaoka, Ralph S. Baric, Blossom Damania, Dirk P. Dittmer

**Affiliations:** 1 Department of Microbiology and Immunology, University of North Carolina at Chapel Hill, Chapel Hill, North Carolina, United States of America; 2 Lineberger Comprehensive Cancer Center, University of North Carolina at Chapel Hill, Chapel Hill, North Carolina, United States of America; 3 Department of Genetics, University of North Carolina at Chapel Hill, North Carolina, United States of America; 4 Department of Epidemiology, University of North Carolina at Chapel Hill, North Carolina, United States of America; 5 Department of Pathobiological Sciences, School of Veterinary Medicine, University of Wisconsin-Madison, Madison, Wisconsin, United States of America; Cardiff University, UNITED KINGDOM

## Abstract

The p53 transcription factor plays a key role both in cancer and in the cell-intrinsic response to infections. The ORFEOME project hypothesized that novel p53-virus interactions reside in hitherto uncharacterized, unknown, or hypothetical open reading frames (orfs) of human viruses. Hence, 172 orfs of unknown function from the emerging viruses SARS-Coronavirus, MERS-Coronavirus, influenza, Ebola, Zika (ZIKV), Chikungunya and Kaposi Sarcoma-associated herpesvirus (KSHV) were *de novo* synthesized, validated and tested in a functional screen of p53 signaling. This screen revealed novel mechanisms of p53 virus interactions and two viral proteins KSHV orf10 and ZIKV NS2A binding to p53. Originally identified as the target of small DNA tumor viruses, these experiments reinforce the notion that all viruses, including RNA viruses, interfere with p53 functions. These results validate this resource for analogous systems biology approaches to identify functional properties of uncharacterized viral proteins, long non-coding RNAs and micro RNAs.

## Author summary

New viruses are constantly emerging. The ORFEOME project was based on the hypothesis that every virus, regardless of its molecular makeup and biology should encode functions that intersect the p53 signaling network, since p53 guards the cell from genomic insults, of which depositing a foreign, viral nucleic acid is one. The result of the ORFEOME screen of proteins without any known function, of predicted open reading frames and of suspected non-coding RNAs is the identification of two viral proteins that interact with p53. The first one, orf10, is encoded by Kaposi Sarcoma-associated herpesvirus and the second one, NS2A, is encoded by the Zika virus.

## Introduction

**Fig 1:**
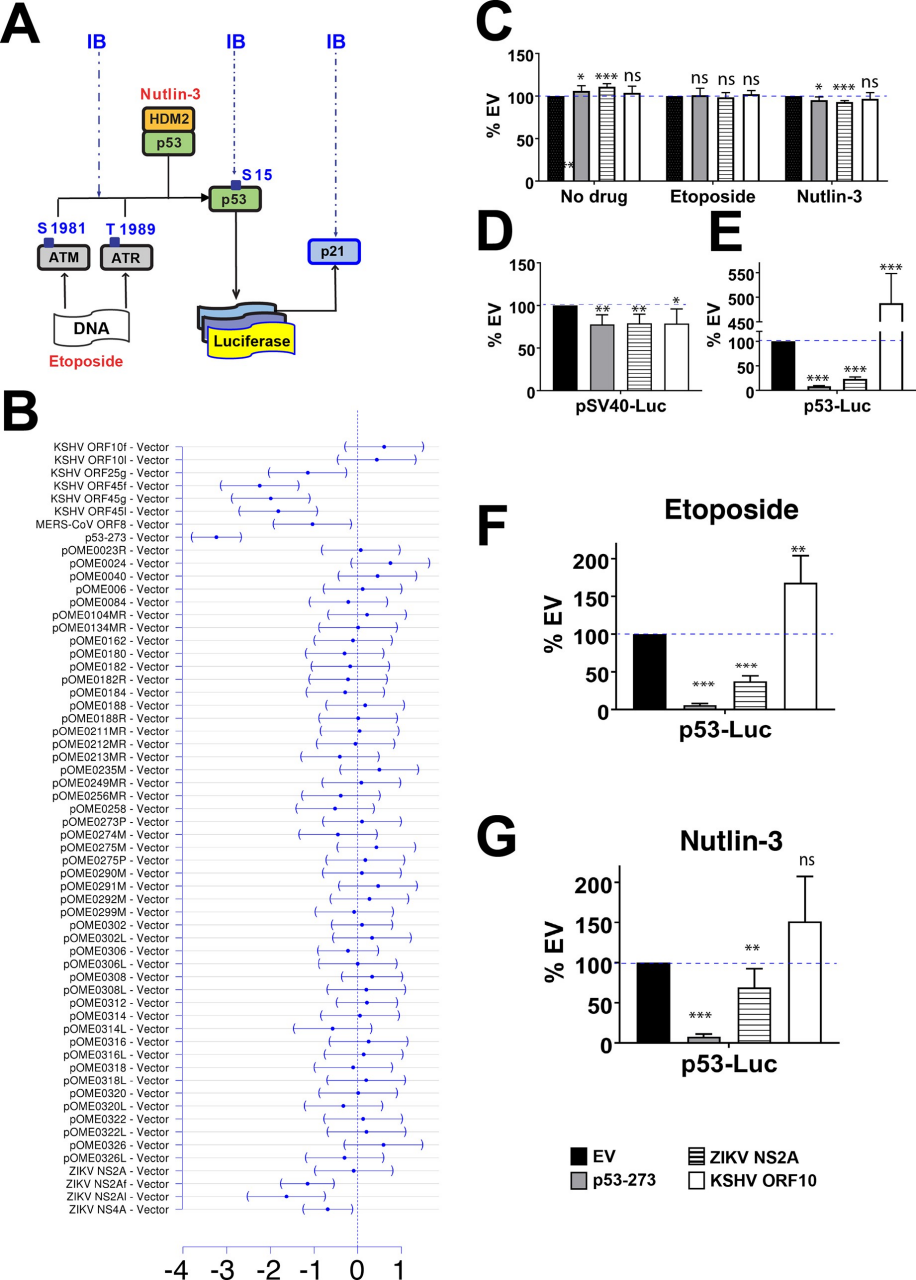
p53-Luc screening of virus encoded orfs. (A) p53 signaling is activated either by (i) etoposide or (ii) nulin-3. Etoposide binds and inhibits topoisomerase II, which results in formation of DSB and activates DDR. ATM senses DSB and autophosphorylates. Because SSB are being introduced during DSB repair, etoposide also activates ATR kinase. Both kinases phosphorylate p53 and HDM2, an E3 ubiquitin ligase, which breaks their interactions and leads to p53 accumulation and stabilization and results in expression of p53 target genes. Nultin-3 inhibits HDM2:p53 interactions and thereby induces p53 accumulation. The response is measured using Luc-reporter assay and immunoblotting with Abs specific for ATM pSer1981, ATR pThr1989, p53 pSer15, p53 and p21. (B) Relative expression levels of p53-dependent luciferase reporter. Shown is the mean effect size plus 95%CI after adjustment for multiple comparisons to control using Dunnett’s methods. The mean effect was calculated by linear model of three independent biological replicate transfections/treatments. For each transfection, three technical replicates were measured. Orfs with confidence intervals not overlapping “0” were considered significant hits (p≤0.05). The names of each tested orf are shown on the left. For detailed description see Experimental Procedures and S1 Table. KSHV orf10f (3xflag-tagged, pOME0004), KSHV orf10l (lentivirus, pOME0004L), KSHV orf45f (3xflag-tagged, pOME0016), KSHV orf45l (lentivirus, pOME0016L), MERS-CoV orf8b (pOME0215), ZIKV NS2A (pOME0303R), ZIKV NS2Af (3xflag-tagged, OME0304), ZIKV NS2Al (lentivirus, pOME0304L). For KSHV orf25g and KSHV orf45g see Experimental Procedures. (C) Cell viability for EV (pCMV-Neo-Bam), p53-273 (pCMV-Neo-Bam- *p53*R273H), ZIKV NS2A (pOME0304), or KSHV orf10 (pOME0004) was measured with CellTiter-Glo luminescence cell viability assay kit following transfection with each vector (18h), treatment with indicated drugs (6h), and additional 24h-incubation. P-values calculated using Student t-test (n = 4) are shown as ‘ns’ (P>0.05), * (P≤0.05), **(P≤0.01), or *** (P≤0.001). (D-G) p53-Luc assays for ZIKV NS2A and KSHV orf10 expressing cells, untreated or stimulated with etoposide or nutlin-3. U2OS cells were transfected with either pGL3 control reporter (D) or p53-responsive reporter pGL13 (E-G) and EV, *p53*R273, ZIKV NS2A-Flag, or KSHV orf10-Flag. At 18 hrs post transfection (p.t.), the cells were stimulated with 5 μM etoposide (F) or 10 μM nutlin-3 (G) for 6h or left untreated (D and E) and then incubated for 24h. Firefly luciferase levels were measured with One-Glo luciferase assay system (Promega Inc.).

The p53 tumor suppressor protein is a transcription factor. It has been called the “guardian of the genome” and it plays major roles in the maintenance of genomic stability, DNA repair, cell cycle arrest, apoptosis, and cell differentiation (reviewed in [1]). Its role in innate immunity is equally established, but less appreciated (reviewed in [2,3]). Normally, p53 protein expression is low and tightly regulated by its interactions with E3 ubiquitin ligases, primarily mouse double minute 2 and its human homolog, human double minute 2 (HDM2). These interactions lead to p53 ubiquitination and degradation via the proteasome pathway. Upon induction of the DNA damage response (DDR) pathway, the DNA damage-sensing kinases, ataxia telangiectasia mutated kinase (ATM), Rad3-Related kinase (ATR), and DNA protein kinase phosphorylate downstream proteins, including HDM2 and p53. This phosphorylation event disrupts the HDM2-p53 interaction, resulting in p53 stabilization, phosphorylation, accumulation, cytoplasmic to nuclear re-localization, and subsequent transcription of p53 target genes, such as p21 (Fig 1A). Any one of these steps poses a target for viral proteins to interrupt p53 signaling and thus premature apoptosis of the infected cell.

Many viruses induce the DDR. The DDR and p53 play dominant roles in the detection of incoming DNA virus genomes (reviewed in [4,5]). P53 also has demonstrated roles in the innate immune response to RNA viruses. Firstly, the p53 promoter contains several interferon response elements. Hence, p53 transcription can be induced by type I interferons (IFNs) [6]. Secondly, p53 expression augments the type I IFN response by inducing interferon regulatory factor 9, interferon-stimulated gene 15, toll-like receptor 3, and protein kinase R (PKR) [7–10]. Lastly, p53 is a target of PKR and is phosphorylated by PKR at Ser392, a site that is important for tetramer stabilization and transcriptional activity [11]. In sum, p53 function is regulated by innate immune signaling and p53 can modulate interferon signaling.

It is not surprising, then, that many viral proteins counteract p53 signaling by a multitude of mechanisms. These include directly interacting with p53, preventing p53 nuclear translocation and/or inducing its degradation, or modulating the activity of other proteins within the p53 signaling network [4,5,12,13]. In fact, p53 was discovered through its binding to the trifecta of DNA virus transforming proteins SV40 large T antigen, adenovirus E1B protein, and high risk-type human papillomavirus E6 protein. These binding events abrogate the activation of p53-dependent promoters. Other viruses interfere with the initial steps of p53 activation by the DDR. Re-localization and inhibition of ATM and ATR and/or components of MRE11-RAD50-NBS1 (MRN) protein complex that are required for ATM and ATR activation have been described for DNA viruses such as adenoviruses, papillomaviruses, polyomaviruses, poxviruses, and herpesviruses, as well as for RNA viruses such as retroviruses, flaviviruses, coronaviruses, and bunyaviruses [4,5,14]. Hepatitis C virus (HCV) and vaccinia virus (VACV), which replicate exclusively in the cytoplasm, induce cytoplasmic accumulation of ATM and ATM/ATR and depend on these proteins for replication [15,16]. HCV and VACV also encode proteins that inhibit downstream signaling by ATM. HCV NS2 and VACV B1R sequester p53 in the cytoplasm and promote its degradation [17,18]. SARS orf6 blocks karyopherin-dependent import of p53 and other transcription factors into the nucleus [19] and p53 interferes with SARS replication and is itself targeted by the “SARS-unique domain and PLpro via E3 ubiquitin ligase” RCHY1 for degradation [20,21]. Some viruses exploit p53-mediated responses to increase efficiency of virus replication or to induce transcription of viral genes [2,12]. Depending on the cellular environment and stage of viral life cycle, p53 signaling is activated or inhibited by viral proteins of almost every known virus.

The ORFEOME project (https://www.med.unc.edu/orfeome) was conceived to systematically identify the biological functions of previously uncharacterized, unknown or hypothetical open reading frames (orfs) and noncoding RNAs across several emerging and reemerging virus families. These were coronaviruses (CoVs), such as severe acute respiratory syndrome (SARS)-CoV, Middle Eastern respiratory syndrome (MERS)-CoV, ancestral bat CoVs, influenza A viruses (IAV), such as highly pathogenic H5N1 avian viruses, 1918 “Spanish” flu, and seasonal H1N1 and H3N2 IAVs, Ebola virus (EBOV), Zika virus (ZIKV), Chikungunya Virus (CHIKV), and Kaposi Sarcoma-associated herpesvirus (KSHV) (reviewed in [22]). The overarching hypothesis of the ORFEOME project was that since all viruses engage p53, one or more of these uncharacterized viral orfs may affect p53 function. To test this hypothesis, a p53-luciferase (p53-Luc) reporter-based screen of 172 orfs encoding viral proteins, micro RNAs (miRNAs), and long non-coding RNAs (lncRNAs) was performed. This screen identified several orfs that were previously unknown to modulate p53 signaling. The two most dramatic hits, ZIKV NS2A, which negatively regulated p53 signaling, and KSHV orf10, which positively regulated p53 signaling, were further characterized to determine their mechanisms of action. These findings reinforce the importance of the p53 pathway in the innate immune response to ZIKV and KSHV infection.

## Results

### Identification of novel modulators of p53 signaling

A library of expression vectors encoding 172 orfs from seven RNA viruses (IFA, EBOV, MERS-CoV, *SARS-CoV*, bat CoV, ZIKV, and CHIKV) and one DNA virus, KSHV (S1 Table), was screened for the ability to change p53-dependent transcription. All plasmids were synthesized *de novo*. Expression of the designed proteins, miRNAs, and lncRNAs was confirmed by immunoblotting, or real-time RT-QPCR. The quality control data, plasmid maps, and sequences are available through the ORFEOME website (https://www.med.unc.edu/orfeome).

The screen used a plasmid encoding the firefly luciferase reporter gene under a promoter containing repeated p53 response elements [23]. This reporter was transfected together with the orf expression plasmids. A plasmid expressing wild-type human p53 was used as a negative control (no inhibition of p53). A plasmid expressing the dominant-negative p53 R273H mutation, which abrogates DNA-binding, but still allows tetramerization [24,25]–further referred to as p53-273 –was used as a positive control for all assays (complete inhibition of p53). The screen was conducted using human bone osteosarcoma epithelial (U2OS) cells [26], which encode wild-type human p53, and in which p53 can be induced with etoposide, a topoisomerase II inhibitor [27]. Etoposide treatment of U2OS cells introduces double-stranded DNA breaks (DSBs) that activate ATM as well as ATR, as DSB repair also involves the formation of single-stranded breaks (SSBs) [28–31]. The screen was repeated in three biological replicates, each one month apart and each transfection conducted in technical triplicates. Each orf was tested as a FLAG-tagged or untagged protein. Each non-coding RNA was expressed from either a CMV-promoter or, in case of miRNAs, from a lentiviral vector. The assay readout, Log10 (Relative luciferase activity), was normally distributed (S1 Fig). Four of the 172 candidate plasmids (2.3%) induced general toxicity as assayed by cell viability in HEK 293 cells and by inhibition of luciferase expression under control of a constitutive promoter from the SV40 virus (S1 Fig). As mentioned above each orf expression plasmid was validated for protein expression by Western-Blot and cellular localization confirmed by immune fluorescence using anti-FLAG antibody. Both FLAG-tagged and untagged expression clones were synthesized and evaluated.

The screen identified five initial hits after adjustment for multiple comparisons (Fig 1B). Hit one was the KSHV orf25, which was previously identified as a p53-inhibiting viral protein [32]. Finding KSHV orf25, which was not nearly as potent a p53 inhibitor as the tumor-derived dominant negative p53-273 mutant, validated the sensitivity of the screen, and conversely KSHV orf25 as a p53 interacting viral protein. Hit two, MERS-CoV orf8b, showed more variable results upon follow-up and was not further characterized. Hit number three, KSHV orf45, is a KSHV immediate early protein that is also part of the virion and interferes with the innate response to KSHV at multiple levels [33]. It is the subject of a separate report.

ZIKV NS2A and KSHV orf10 emerged as the most reproducible regulators of p53 that had not been implicated in p53 signaling before. ZIKV NS2A inhibited and KSHV orf10 augmented the p53-mediated transcriptional response to etoposide. To validate these candidates, we proceeded as follows: first, we tested whether ZIKV NS2A and KSHV orf10 showed toxicity by conducting cell viability assays (Fig 1C–1G) in the absence of drug, or in the presence of etoposide, or in the presence of nutlin-3, a small molecule inhibitor that binds to HDM2 and blocks its interactions with p53, thereby activating p53 signaling [34]. ZIKV NS2A and KSHV orf10 had no effect on overall cell viability under either scenario. Second, we tested whether these orfs affected basal promoter activity non-selectively by using a plasmid constitutively expressing luciferase under the SV40 promoter (SV40-Luc, Fig 1D). ZIKV NS2A and KSHV orf10 had no effect on basal promoter activity. Third, we again verified that these orfs efficiently downregulated (ZIKV NS2A) or upregulated (KSHV orf10) p53-driven expression of luciferase (p53-Luc) in response to etoposide. In addition, we tested whether ZIKV NS2A and KSHV orf10 also had an effect on p53 in the absence of the drug. ZIKV NS2A and KSHV orf10 strongly affected p53-Luc expression in etoposide-stimulated cells, as well as in untreated cells (Fig 1E and 1F). ZIKV NS2A interfered with endogenous p53 transactivation as efficiently as p53-273, a dominant-negative mutant. KSHV orf10-induced p53-Luc ~5-fold compared to empty vector (EV) in mock-treated cells. The effect of KSHV orf10 was less pronounced in etoposide-treated cells, as here the highly induced p53 likely saturated the reporter response.

To understand at which stage p53 signaling was inhibited, these orfs were evaluated in U2OS cells stimulated with nutlin-3, a HDM2-p53 interaction inhibitor (Fig 1G). ZIKV NS2A inhibited p53 transcription induced by nutlin-3, but less efficiently than the dominant-negative p53-273 mutant, suggesting that ZIKV NS2A may interfere with DDR-induced activation of p53 as well as the signaling downstream of HDM2. KSHV orf10 again augmented the response to nutlin-3 compared to EV, suggesting that it acted directly on p53. These experiments verified the results of the primary screen and identified two novel viral modulators of p53 signaling, potentially using novel molecular mechanisms.

### ZIKV NS2A interferes with p53 nuclear localization

**Fig 2:**
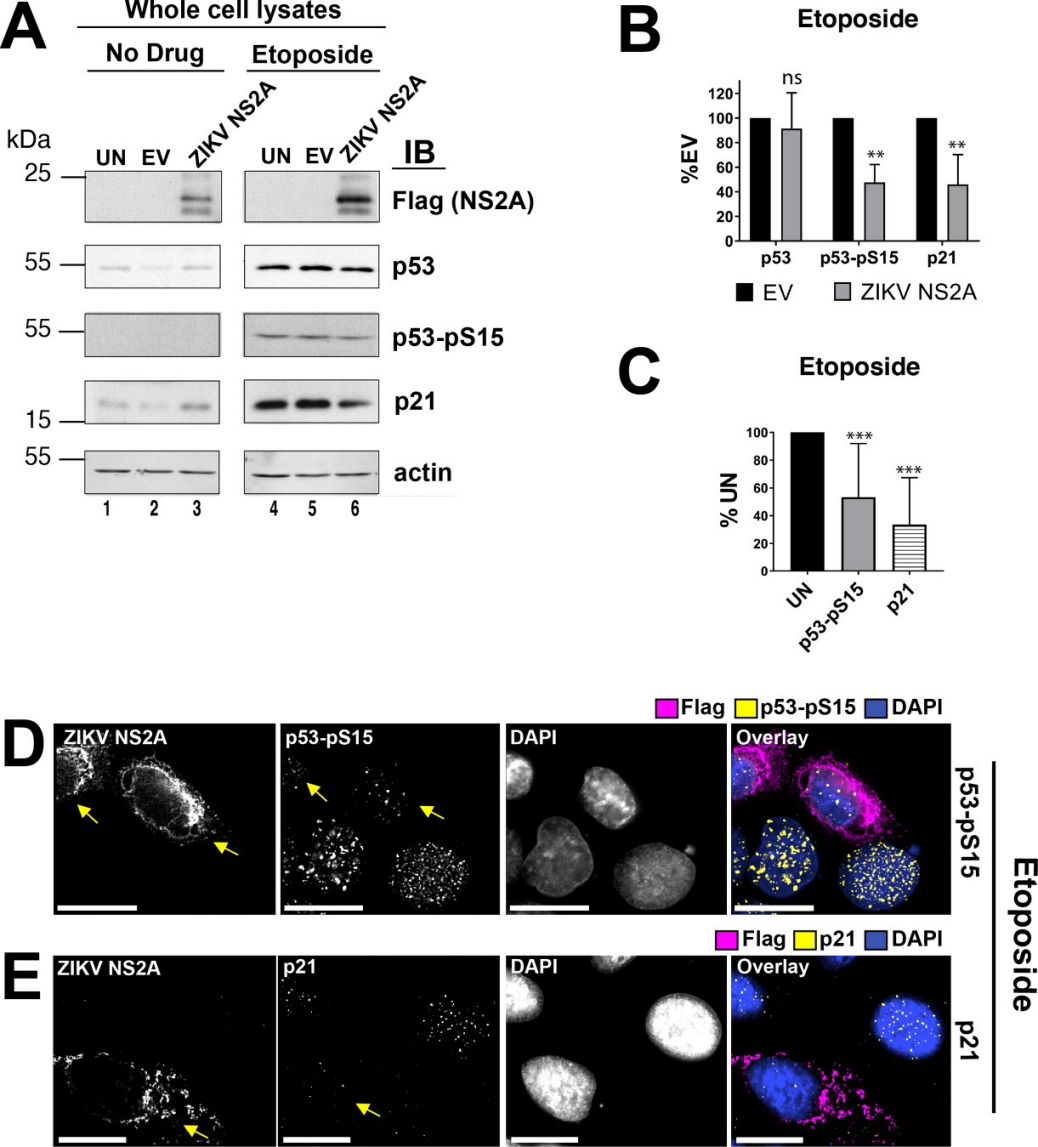
Expression of the p53-signaling components in the presence of ZIKV NS2A. (A) Cells were transfected with empty vector (EV) and pOME0303R (ZIKV NS2A) or left untransfected (UN). At 18h after transfection, the cells were stimulated with 10 μM etoposide for 6h. The cell lysates were analyzed by SDS-PAGE and immunoblotting with indicated antibodies. (B) Shows protein levels by immunoblotting (IB) normalized to beta-actin relative to empty vector (% EV) for three different experiments that were calculated based on pixel density of each protein band measured using ImageJ software. Bars indicate the standard error of mean (s.e.m., n = 3). P-values calculated using Student t-test are shown as ‘ns’ (P>0.05), * (P≤0.05), **(P≤0.01), or *** (P≤0.001). (C) Shows protein levels in ZIKV NS2A expressing cells by immunofluorescence (IF) relative to untransfected cells (%UN) that were calculated based on pixel density measured using ImageJ software. Bars indicate s.e.m. (n≥10). P-values calculated using Student t-test are shown as ‘ns’ (P>0.05), * (P≤0.05), **(P≤0.01), or *** (P≤0.001). (D and E) U2OS cells were transfected with ZIKV NS2A-Flag. At 18h after transfection the cells were stimulated with 10 μM etoposide for 1.5 hrs, fixed, and stained with anti-Flag, anti-phospho-p53Ser15, or anti-p21 antibodies. DAPI was used to delineate the nucleus. The images were subjected to a digital deconvolution. Each image represents an individual optical section. The scale bar is 50 μm. Arrows point at the cell expressing ZIKV NS2A.

To investigate the mechanism of action for ZIKV NS2A, three key biochemical markers of p53 signaling were evaluated: p21 expression, p53 nuclear localization and p53 Ser15 phosphorylation. Ser15 phosphorylation of p53 (p53-pS15) is an established marker of DDR-dependent induction of p53 and essential for p53-dependent transactivation. U2OS cells were transfected with a plasmid expressing Flag-tagged ZIKV NS2A and then stimulated with etoposide for 6h. Total cell lysates were analyzed by SDS-PAGE and immunoblotting (Fig 2A). As expected, etoposide robustly induced p53 and p53-Ser15 phosphorylation. ZIKV NS2A-Flag protein migrated at the predicted molecular weight (~26.8 kDa), but lower molecular weight species migrating at ~20 kDa were also detectable. Observing multiple bands for NS2A was consistent with prior studies [35]. At this point, it is unclear if the ZIKV NS2A undergoes proteolytic processing or if there were additional ZIKV NS2A isoforms expressed from internal start codons. Expression of ZIKV NS2A did not affect the accumulation of total and phosphorylated p53 by immunoblotting (90% and 50% of EV, respectively) (Fig 2A and 2B). Next, we measured levels of p21 by immunoblotting and immunofluorescence. There was an increase in p21 accumulation in the presence of ZIKV NS2A in untreated cells, which suggested that ZIKV NS2A may play a role in regulating p53 independent p21 expression. In etoposide-treated cells, ZIKV NS2A slightly decreased the accumulation of p21 (Fig 2A, quantitation in Fig 2B). The magnitude of the effect, as determined by Western-Blot, was less than ideal and can be explained as follows. First, the transfection efficiency of U2OS is comparatively low. Second, not all cells respond to etoposide and induce p53. For instance, cells in G2 phase of the cell cycle do not respond to DNA damaging drugs and cells actively replicating DNA (S phase) respond more dramatically than resting cells (G0/G1).

To overcome this limitation, the experiment was repeated, and analysis conducted at the single-cell level. As p53-pS15 is an immediate modification, U2OS cells transiently expressing ZIKV NS2A-Flag fusion protein were stimulated with etoposide for only 1.5 hours before analysis. ZIKV NS2A reduced p53-pS15 accumulation only in transfected cells (Fig 2C and 2D). As expected, ZIKV NS2A-Flag localized in the cytoplasm, next to endoplasmic reticulum (ER) and Golgi. Furthermore, ZIKV NS2A expression significantly decreased nuclear accumulation of p21 in etoposide-treated cells by immunofluorescence (shown by arrows, Fig 2E, quantitation in Fig 2C). These experiments establish ZIKV NS2A as a novel viral regulator of p53.

### ZIKV NS2A binds to p53

**Fig 3:**
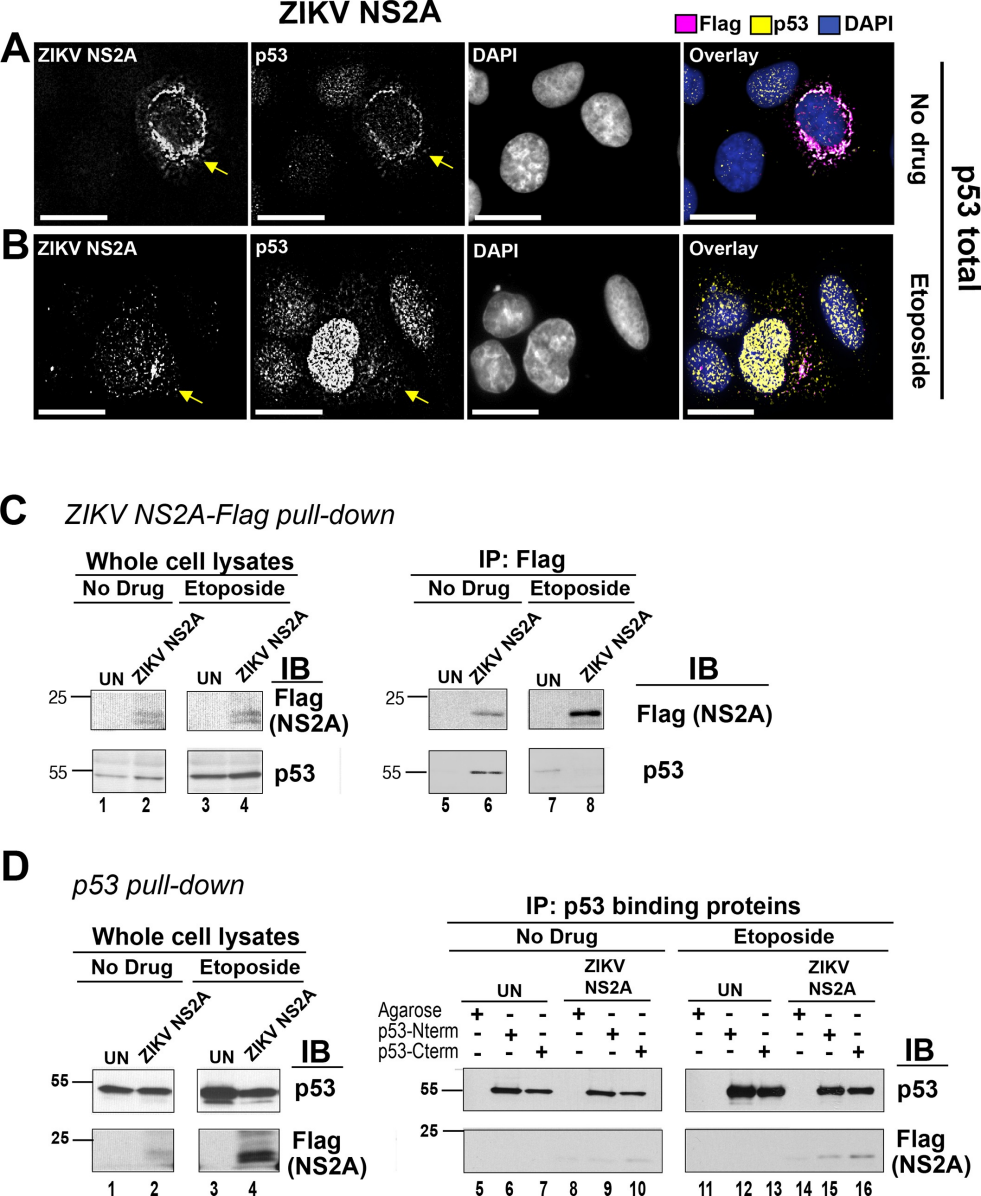
ZIKV NS2A binds to p53. (A, B) U2OS cells were transfected with ZIKV NS2A-Flag. At 18h p.t., the cells were left untreated (A) or stimulated with 10 μM etoposide for 1.5 hrs (B), fixed with methanol and stained with indicated Abs. The images were taken as Z-stack sections and subjected to a digital deconvolution. The scale bar is 50 μm. Arrows point at the cell expressing ZIKV NS2A. (C) Endogenous p53 pull-down. U2OS cells were transfected with pDEST47-ZIKV NS2A-Flag expressing ZIKV NS2A tagged with the C-terminal 3xFlag epitope or left untransfected (UN). At 18 hrs p.t., the cells were stimulated with 10 μM etoposide for 6 hrs. NS2A-Flag was immunoprecipitated with mouse α-Flag Ab. Lysates and imunoprecipitates was tested with mouse mab α-p53 DO7 and mouse α-Flag Abs. (D) ZIKV NS2A pull-down using p53-binding proteins. U2OS cells were transfected with pDEST47-ZIKV NS2A-Flag or left untransfected (UN). At 18 hrs p.t., the cells were stimulated with 10 μM etoposide for 6h. P53 was immunopreciptated with proteins binding to p53 N- or C-terminus. Presence of ZIKV NS2A-Flag and p53 in the lysates and imunoprecipitates was tested with mouse anti-Flag and mouse α-p53 DO7 Abs.

In the absence of etoposide, the immunofluorescence signals for ZIKV NS2A and p53 overlapped (Fig 3A). Total p53 showed the same perinuclear ring staining as ZIKV NS2A. The colocalization was less pronounced in the presence of etoposide. Some p53 still entered the nucleus (Fig 3B) and was carrying the activating Ser15 phosphorylation, as shown in Fig 2D.

To verify this phenotype in a second cell line with established biological relevance for ZIKV, yet still containing wild-type p53, the experiments were repeated in U87MG glioma cells [36]. Here, the results were more pronounced. p53 colocalized with ZIKV NS2A in the cytosol of transfected cells in the absence as well as in the presence of etoposide (shown by arrows, S2A and S2B Fig).

The colocalization of ZIKV NS2A with p53 suggested that these two proteins interacted with each other. To test this hypothesis, the ZIKV NS2A-Flag was transfected into U2OS cells and protein-protein interaction was probed by co-immunoprecipitation. Expression of ZIKV NS2A-Flag and endogenous p53 was readily detectable (Fig 3C, left). Next, α-Flag antibody was used to immunoprecipitate ZIKV NS2A-Flag, and the blot was probed for p53. p53 co-immunoprecipitated with ZIKV NS2A in the absence of etoposide treatment, but not in the presence of etoposide treatment (Fig 3C, right). This result confirmed the immunofluorescence data and is consistent with a model that in etoposide-treated cells, p53 is stabilized and not all of the p53 is available for ZIKA NS2A binding.

Some technical aspects of this experiment deserve attention. First, we were unable to procure or produce a NS2A antibody with satisfactory performance in Western-Blot and immunoprecipitation experiments. Second, a high-stringency wash buffer (500 mM NaCl, 1% NP-40) was used to counteract the non-specific binding of ZIKA NS2A to beads. This may have reduced the sensitivity of the binding assay. To address these limitations, a reverse co-immunoprecipitation experiment was conducted. Exceptionally sensitive and specific reagents are available for p53. Small, high-affinity p53-binding proteins (α-p53 VHH) directed against either the C- or the N-terminus of p53 and covalently coupled to agarose beads were used for immunoprecipitation. Subsequently the precipitates were resolved by SDS-Page, transferred to polyvinylidene difluoride membranes and probed for ZIKV NS2A-Flag with α-Flag antibody. ZIKV NS2A from untreated as well as etoposide-stimulated samples reliably coprecipitated with p53 (Fig 3D, lanes 10, 15, 16). These experiments demonstrate that ZIKV NS2A interacts with p53.

### ZIKV NS2A affects ATM accumulation and/or localization

**Fig 4:**
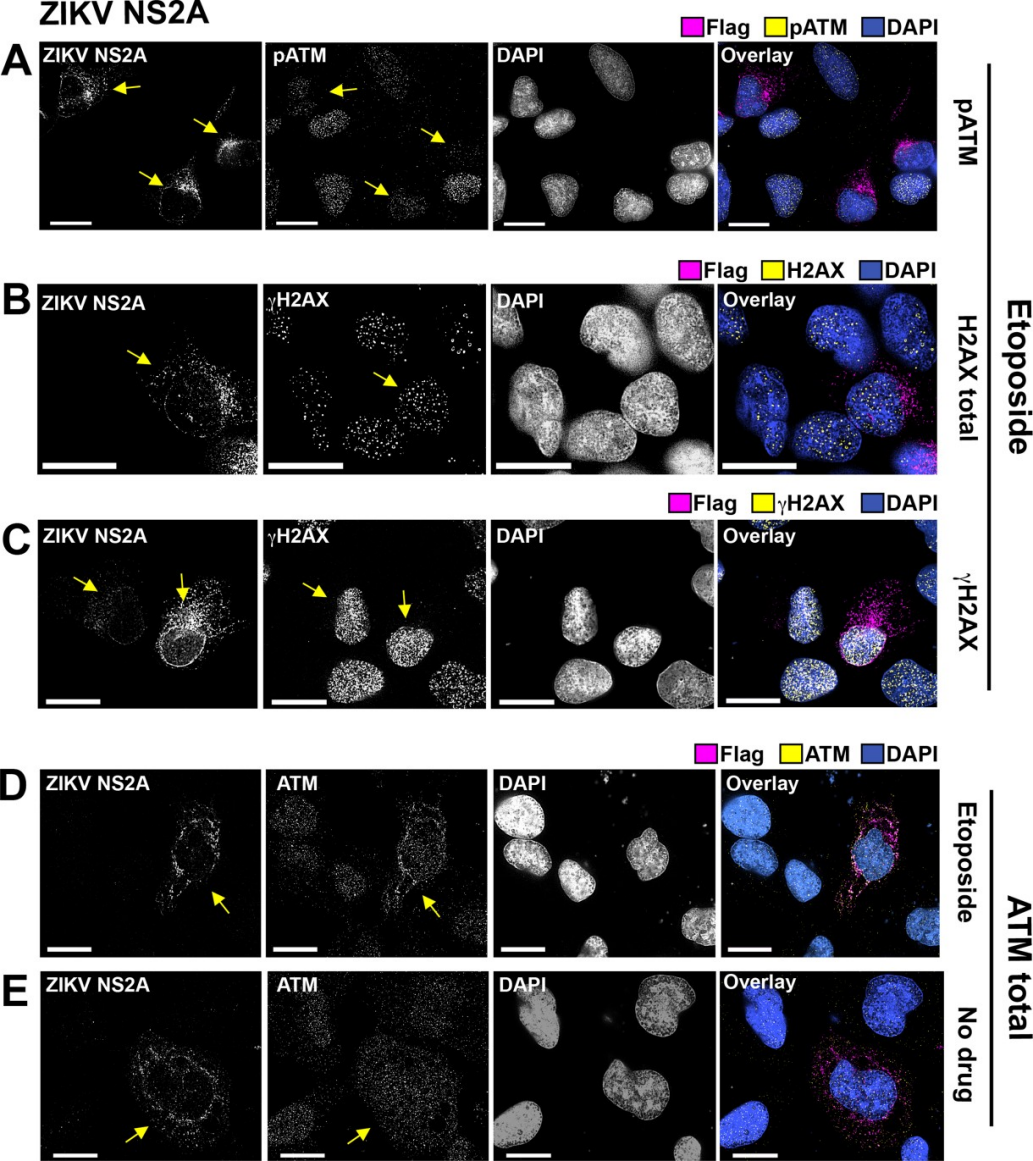
Localization of ATM and pATM, H2AX, and μH2AX in the presence of ZIKV NS2A. U2OS cells were transfected with ZIKV NS2A-Flag (pOME0304). At 18 hrs p.t., the cells were stimulated with 10 μM etoposide for 1.5 hrs (A, C-E) or left untreated (B), fixed with methanol, and stained with indicated Abs. The scale bar is 50 μM. Arrows point at the cell expressing ZIKV NS2A.

ZIKV NS2A bound to p53 and was able to inhibit etoposide-induced activation of a p53-responsive promoter; however, it had a noticeably weaker effect on nutlin-3 -induced activation of p53 (Fig 1). This led to the hypothesis that this protein may also affect the accumulation and/or phosphorylation of ATM or ATR upstream of p53. No changes in the levels of total or phosphorylated ATM or ATR were evident in the presence of ZIKV NS2A when compared to empty vector by Western Blot (S3A Fig) Suspecting that, as outlined above, not all cells were transfected and not all cells responded synchronously to etoposide stimulation, immunofluorescence was used to evaluate individual cells. ZIKV NS2A reduced nuclear ATM phosphorylation in transfected cells (shown by arrows, Fig 4A). In contrast to ATM nuclear accumulation and phosphorylation of ATR was not affected (shown by arrows, S3B–S3D Fig). Total histone gamma-H2AX (γH2AX), a bona fide target of both ATM and ATR [37], was not affected either. We were not, however, able to conclusively assign a direct effect of ZIKV NS2A on ATM localization. ZIKV NS2A increased the cytoplasmic localization of ATM in U2OS cells (shown by arrows, Fig 4D and 4E), but this phenotype was not observed in the U87MG glioma cell line (S2C and S2D Fig). Taken together, these observations suggest that ZIKV NS2A can affect the p53 signaling network at multiple non-exclusive nodes: (i) NS2A binds to p53, it traps p53 in the cytosol, prevents its nuclear translocation, and thereby dampens p53 transcriptional activity; and (ii) it inhibits DDR mediated by ATM under some circumstances.

### KSHV orf10 induces p53 levels, phosphorylation, and nuclear accumulation

**Fig 5:**
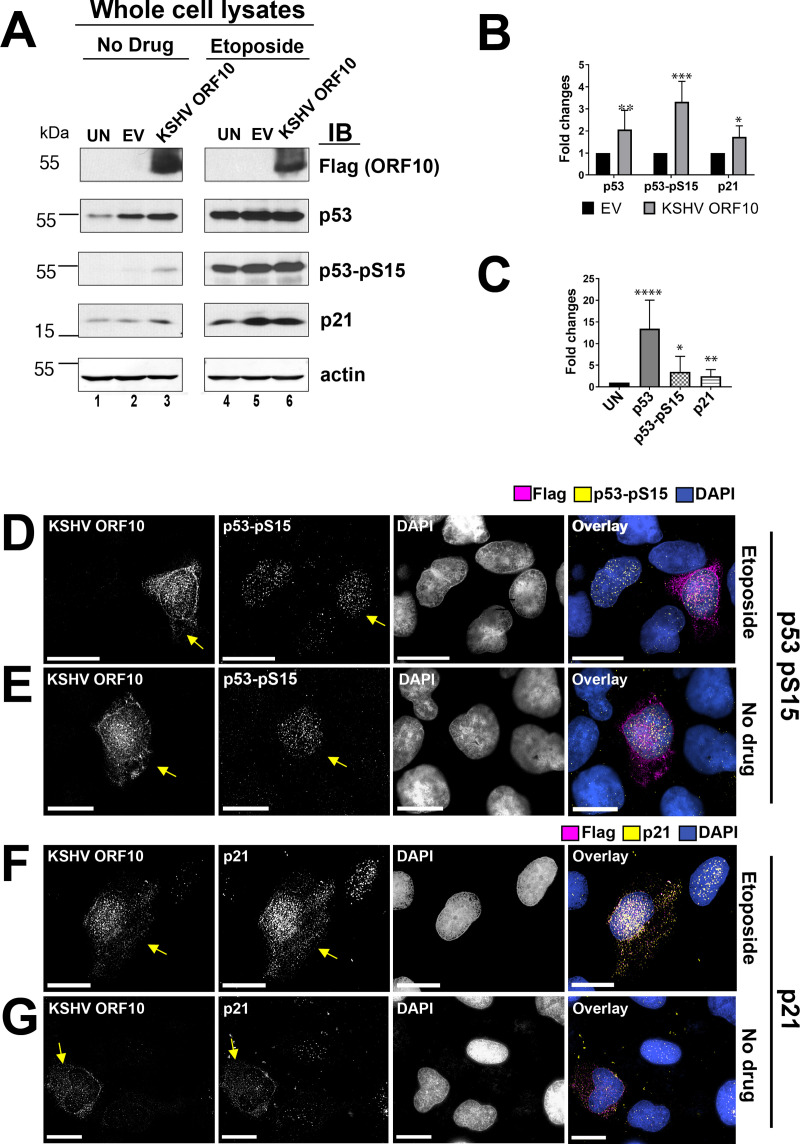
Induction of p53, p53 pSer15, and p21 by KSHV orf10. (A) Cells were transfected with empty vector (EV) and pOME0004 (KSHV orf10) or left untransfected (UN). At 18h p.t., the cells were stimulated with 10μM etoposide for 6h or mock treated (No Drug). The cell lysates were analyzed by SDS-PAGE and immunoblotting with indicated Abs. (B) Shows relative protein expression levels (fold changes compared to EV) by IB cumulative of two different experiments that were calculated based on pixel density of each protein band measured using ImageJ software. Bars indicate the s.e.m. (n ≥ 3). P-values calculated using Student t-test (n = 3) are shown as ‘ns’ (P>0.05), * (P≤0.05), **(P≤0.01), or *** (P≤0.001). (C) Shows protein levels in orf10 expressing cells by IF relative to mock transfected cells (fold changes compared to UN) that were calculated based on pixel density measured using ImageJ software. Bars indicate s.e.m. (n≥10). P-values calculated using Student t-test (n = 3) are shown as * (P≤0.05), **(P≤0.01), or *** (P≤0.001), **** (P≤0.0001). (D-G) U2OS cells were transfected with KSHV orf10-Flag. At 18 hrs p.t., the cells were stimulated with 10 μM etoposide for 1.5 hrs (D, F) or left untreated (E, G), fixed with methanol, and stained with indicated Abs. The scale bar is 50 μM. Arrows point at the cells expressing KSHV orf10.

**Fig 6:**
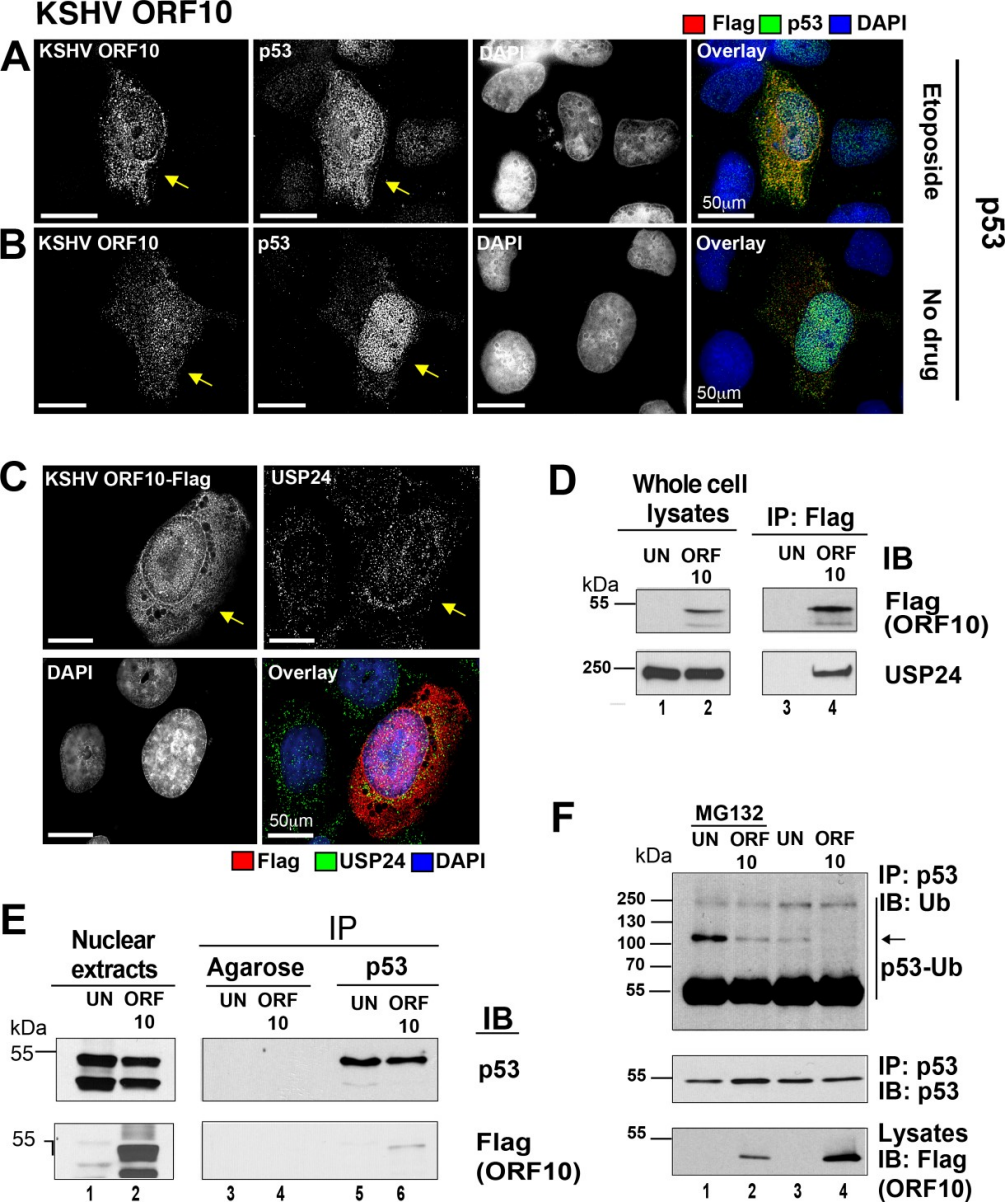
KSHV orf10 interacts with USP24 and p53. (A, B) U2OS cells were transfected with pDEST47-KSHV orf10-Flag. At 18h p.t., the cells were stimulated with 10μM etoposide for 1.5h (A) or left untreated (B), fixed with methanol, and stained with indicated Abs. Each image represents an individual optical section. The scale bar is 50μM. Arrows point at the cells expressing KSHV orf10. (C) KSHV orf10 colocalizes with USP24. U2OS cells, transfected with pDEST47-KSHV orf10-Flag for 18h were fixed with methanol and stained with indicated α-Flag and α-USP24 Abs. Each image represents an individual optical section. The scale bar is 50μM. Arrow points at the cell expressing KSHV orf10. (D) USP24 coimmunoprecipitates with KSHV orf10. U2OS cells were transfected with pDEST47-KSHV orf10-Flag expressing orf10 tagged with C-terminal 3xFlag epitope or left untransfected (UN) for 18h. orf10-Flag was immunoprecipitated from the whole cell lysates with mouse α-Flag Ab. Presence of USP24 and orf10-Flag in the lysates and coimunoprecipitated fractions was tested with rabbit α-USP24 and mouse α-Flag Abs. (E) KSHV orf10 coimmunoprecipitates with p53. U2OS cells were transfected with pDEST47-KSHV orf10-Flag or left untransfected (UN) for 18h. P53 was immunopreciptated from nuclear fraction of cell lysates with proteins binding to p53 N- or C-terminus. Presence of orf10-Flag and p53 in the lysates and coimunoprecipitated fractions was tested with mouse α-Flag and mouse α-p53 DO7 Abs. (F) p53 ubiquitination is reduced in the presence of KSHV orf10. U2OS cells were transfected with KSHV orf10 or left untransfected. At 18h p.t., the cells were incubated in the presence or absence of 30μM MG132 for 6h. p53 was coimmunoprecipitated with p53-specific mouse pAb421 Ab and blotted with rabbit α-ubiquitin and mouse α-p53 DO7 Abs.

KSHV orf10 emerged as a novel enhancer of p53-dependent transcription in this screen (Fig 1). To verify this phenotype, U2OS cells were transfected with a plasmid expressing KSHV orf10 tagged with a C-terminal Flag epitope for 18h and then stimulated with etoposide for 6h. The cell lysates were analyzed by SDS-PAGE and immunoblotting. KSHV orf10-Flag protein migrated at the predicted molecular weight of 48.8 kDa (Fig 5A). As expected, etoposide robustly induced p53 and p53-Ser15 phosphorylation. As compared to NS2A under the same conditions (Fig 2A), KSHV orf10 had the opposite effect. The effect of KSHV orf10 expression on p53 Ser15 phosphorylation compared to EV was around 4-fold in untreated cells (Fig 5A, quantitated in Fig 5B), i.e. KSHV orf10 induced p53 Ser15 phosphorylation independent of etoposide. In the presence of etoposide, p53 Ser15 was already maximally phosphorylated even in mock-transfected cells. Hence, KSHV orf10 had no additional effect. KSHV orf10 also increased accumulation of total p53 levels as measured by Western-Blot of the total cell population. By individual cell analysis, KSHV orf10 expression increased phosphorylated p53 levels (Fig 5D and 5E, and quantitated in Fig 5C), consistent with the Western-Blot results. Furthermore, KSHV orf10 increased the levels of total p53 only in transfected, untreated cells (Fig 6A and 6B, shown by arrows). These experiments confirmed that KSHV orf10 augments p53 signaling in response to DNA damage and that KSHV orf10 stabilizes p53 in the absence of DNA damage signals.

KSHV orf10 colocalized with p53 (Fig 6A and 6B), though this was difficult to ascertain, as both proteins were detectable in the nucleus as well as in the cytoplasm of transfected cells. The effect of KSHV orf10 on p53 was verified in a second cell line of relevance to KSHV biology: human Telomerase-immortalized *H**uman* *U**mbilical* *V**ein* *E**ndothelial* *C**ells (htert-*HUVEC), which also express wild-type p53 protein [38]. The p53 protein was induced and accumulated in the nucleus in *hTert-*HUVEC expressing KSHV orf10, in the presence as well as in the absence of etoposide (shown by arrows, S2E and S2F Fig). The p21 protein was upregulated consistent with complete p53 pathway activation (shown by arrows, Fig 5F and 5G). These experiments establish KSHV orf10 as a new p53 interacting protein encoded by a human tumor virus.

Since KSHV orf10 induced p53 Ser15 phosphorylation in the absence of etoposide, ATM and ATR activation was investigated in untreated U2OS cells expressing the KSHV orf10-Flag fusion protein. As a positive control KSHV orf57/MTA, a viral splice-factor was included because it induces dsDNA breaks and thereby ATM/ATR activation [39]. KSHV orf10 did not increase ATM phosphorylation, nor alter its total levels of expression (S4A Fig). Furthermore, KSHV orf10 had no effect on the accumulation and phosphorylation of ATR and did not result in phosphorylation of γH2AX, suggesting that orf10 acts downstream of ATM and ATR. This mechanism of action is consistent with the ability of KSHV orf10 to augment p53-dependent transcription in response to Nutlin-3 (Fig 1G), i.e., independent of ATM/ATR activation. These results establish KSHV orf10 as a novel modulator of p53 signaling. KSHV orf10 augments p53 levels, phosphorylation, and nuclear localization.

### KSHV orf10 coimmunoprecipitates with p53 and reduces p53 ubiquitination

KSHV orf10 shuttles between the nucleus and the cytoplasm and interacts with nuclear pore proteins such as Nup98 [40]. P53 also shuttles between the nucleus and the cytoplasm. These observations prompted the hypothesis that orf10 interferes with p53 nuclear export because CRM1 is a p53 nuclear export receptor and the CRM1-Nup98 complex is necessary for CRM1-dependent nuclear export [41]. Contrary to expectations, however, KSHV orf10 did not interact with CRM1, nor did KSHV orf10 inhibit CRM1-dependent nuclear export (S4B–S4E Fig). These data necessitated a different model to explain the orf10-enhanced nuclear accumulation of p53.

Earlier, a screen for host factors interacting with KSHV-encoded proteins identified USP24, a deubiquitinase, as a potential partner of orf10 in HEK293T cells [42]. P53 is also a substrate of USP24 [43]. Hence, orf10 could bring USP24 and p53 together, acting as a scaffold protein. To test this hypothesis, the published orf10 and USP24 interaction was verified in p53 wild-type U2OS cells. First, protein colocalization was established. Both proteins colocalized in the cytoplasm, perinuclear space, and the nucleus of transfected cells (Fig 6C). Second, direct binding was verified. Immunoprecipitation of KSHV orf10-Flag fusion from transfected U2OS cell lysates successfully pulled down USP24 (Fig 6D). These experiments confirm the prior reports of the KSHV orf10-USP24 interaction in a second, independent cell line.

Next, we tested for protein-protein interactions between transfected KSHV orf10 and endogenous p53. At present no suitable KSHV orf10 reagents are available. The KSHV orf10-Flag coimmunoprecipitated with p53 using p53-binding protein as high affinity reagent (Fig 6E). Here, the nuclear extract fraction was used as input, as it contained the majority of KSHV orf10 and p53. To explore the functional consequences of this novel interaction, a p53 ubiquitination assay was performed. p53 ubiquitination was reduced in the presence of KSHV orf10 as compared to control (Fig 6F, lane 2 compared to lane 1**)**. As expected, the effect was very pronounced in the presence of the proteasome inhibitor MG132 but was also evident in the absence of MG132 (the arrow points to ubiquitinated species of p53 migrating above 100 kDa, Fig 6F, lane 4 compared to lane 3). This demonstrates that KSHV orf10 can act as a scaffolding protein leading to p53 deubiquitination and stabilization. This mechanism likely explains the increased p53 transcriptional activity in the primary screen.

**Fig 7:**
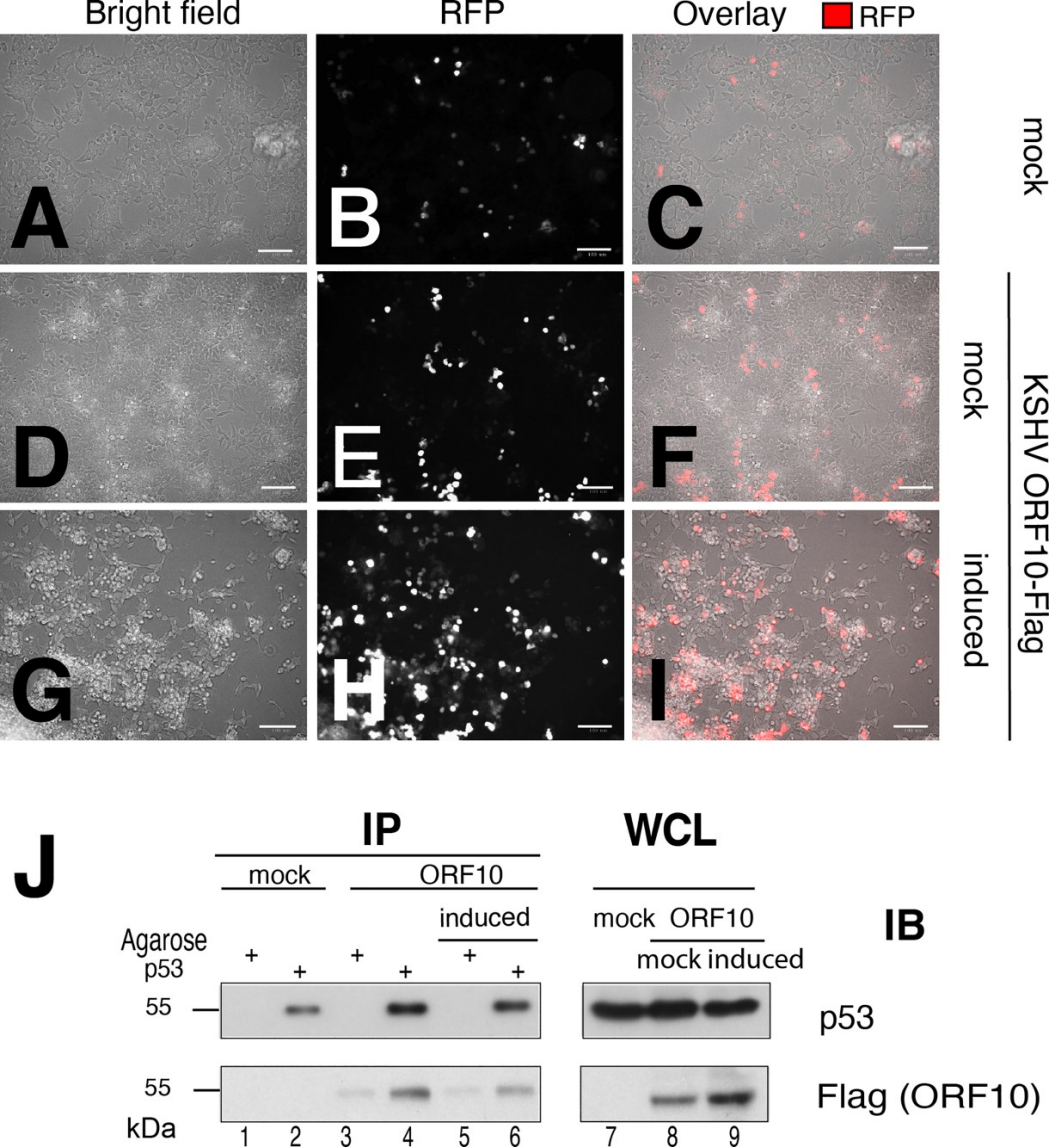
KSHV orf10 interacts with p53 in KSHV infected cells. (A) A293T.219 cells were transfected with pDEST47-KSHV orf10-Flag. At 16h p.t., the cells were induced with 1mM NaB and 25 ng/ml 12-O-tetradecanoylphorbol-13-acetate (*TPA*) for 48h. Stimulation of A293T.219 cells was monitored by RFP expression. Transfected stimulated and unstimulated cells were imaged with bright field and fluorescence microscopy to visualize upregulation of expression of RFP and cytopathic effect (CPE) induced by KSHV replication. Panel A—C show mock-transfected, uninduced cells, panels D—I depict KSHV ORF10-FLAG transfected cells that were either left untreated or induced. Panels (J) KSHV orf10 coimmunoprecipitates with p53. p53 was immunopreciptated from whole cell lysates with antibody binding to p53 C-terminus. Presence of orf10-Flag and p53 in the lysates and co-imunoprecipitated fractions was tested with mouse anti-Flag and mouse anti-p53 DO7 Ab.

Next, we confirmed KSHV orf10-p53 interactions in the context of KSHV lytically infected cell. Because KSHV orf10 specific Ab are not available, HEK293T.219 cells carrying latent KSHV were used. HEK293T.219 carry the 219 strain of KSHV [44]. The virus can be reactivated and cells that undergo complete lytic replication are marked by expression of the red fluorescent protein. The HEK293T.219 cells were transfected with KSHV ORF10-Flag expressing plasmid and stimulated. Progression to lytic cycle was monitored (a) by following expression of the RFP-reporter expressed under the PAN promoter and (b) by observing cytopathic effected (CPE) induced by virus replication (Fig 7A–7I). Under these conditions KSHV orf10 coimmunoprecipitated with p53 pulled down using p53–specific Ab (Fig 7G). This demonstrates that in the presence of all the other known p53-interacting KSHV proteins the KSHV orf10-p53 complex is still formed.

### KSHV orf10 requires USP24 for upregulation of p53 expression and phosphorylation

**Fig 8:**
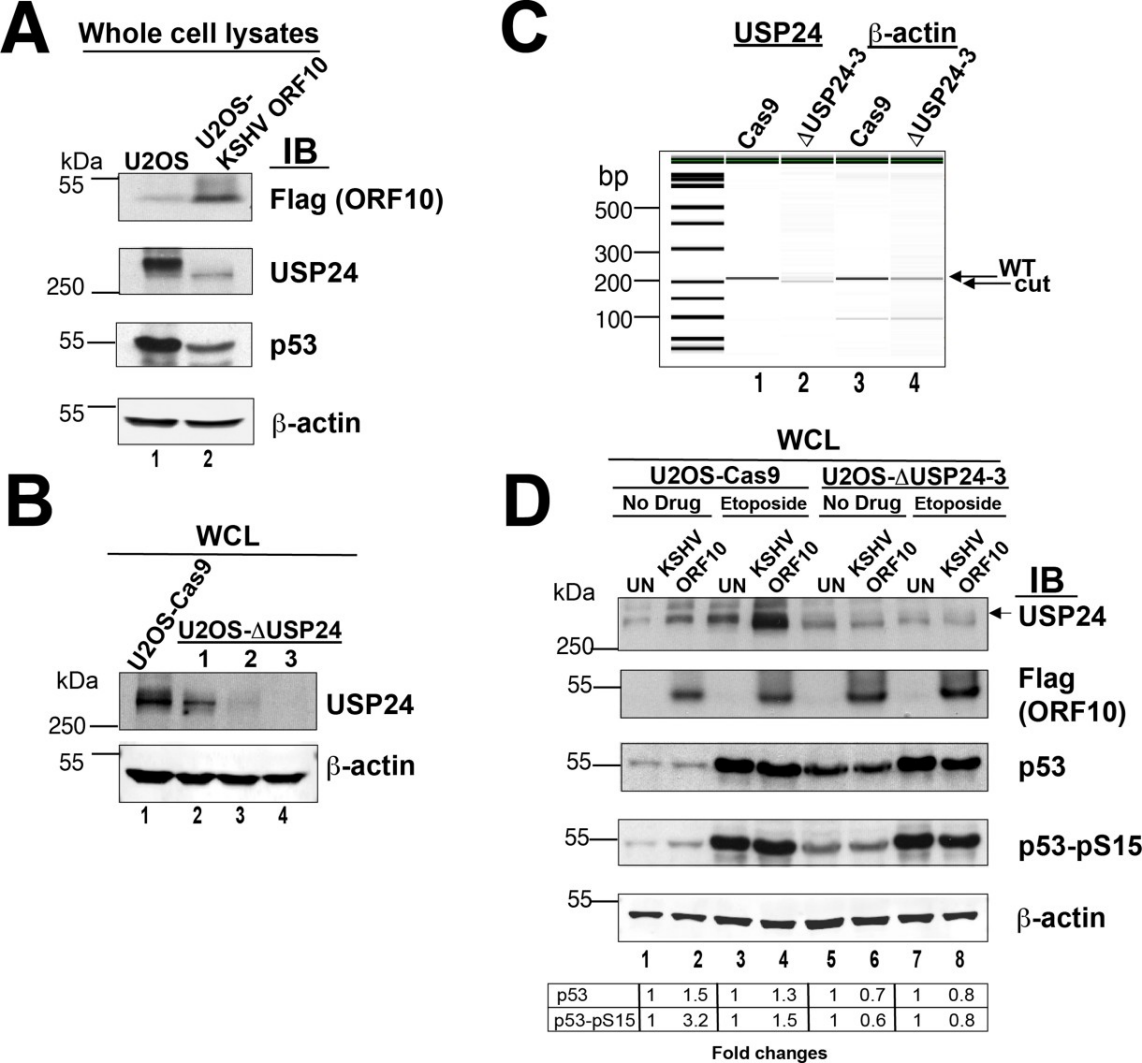
KSHV orf10 does not upregulate accumulation and phosphorylation of p53 in in the absence of USP24. (A) U2OS cells stably expressing KSHV ORF10 show decreased expression of USP24 and p53. Lysates of U2OS cells and U2OS-KSHV ORF10 cells stably expressing KSHV ORF10-Flag were analyzed by SDS-PAGE and immunoblotting with indicated antibodies. (B) Testing USP24 expression in U2OS USP24 KO cell lines. Lysates U2OS-Cas9 parental cell line or indicated U2OS-ΔUSP24 cell lines were analyzed by SDS-PAGE and immunoblotting with indicated Abs. (C) Analysis of CRISPR/Cas-9 deletion of USP24. Shown is an image of capillary electrophoresis of PCR products of DNA using primers flanking the CRISPR target site and actin as control. (D) KSHV ORF10 expression in USP24 KO cells does not induce accumulation and phosphorylation of p53. U2OS-Cas9 or U2OS-dUSP24-3 cells were transfected with pDEST47-KSHV orf10-Flag or left untransfected (UN). At 18h p.t., the cells were stimulated with 10 μM etoposide for 6h or mock treated (No Drug). The cell lysates were analyzed by SDS-PAGE and immunoblotting with indicated Abs.

To explore the role of USP24 in KSHV orf10 mediated p53 stimulation more in depth, we generated U2OS cells stably expressing KSHV orf10-Flag. These cells displayed significantly decreased levels of not only p53, but also USP24 (Fig 8A), suggesting that forced, long-term expression of orf10 has a profound effect and resets the equilibrium or steady state levels of both p53 and USP24. This third, independent assay format thus supports a direct interaction between USP24, p53 and orf10.

Next, we deleted USP24 gene in U2OS cells using CRISPR/Cas9, which generated three independent US2OS-ΔUSP24 cell populations upon long-term selection. The US2OS-ΔUSP24-3 cell line had virtually undetectable USP24 by immunoblotting (Fig 8B**)**. The other two cell populations were not as pure. Quantitative PCR analysis of clone 3 using primers surrounding the deletion region showed the USP24-specific fragment to be slightly smaller compared to the WT USP24 (Fig 8C), which is indicative of a deletion. Thus, the PCR analysis was consistent with the protein analysis. Both sets of experiments demonstrated that USP24 expression was abolished and that U2OS cells deleted for USP24 are viable, most likely because loss of USP24 can be compensated for by cellular reprogramming or the presence of other, compensating USPs in the cell.

Next, we tested p53 expression and phosphorylation in KSHV orf10 transfected cells. In the absence of etoposide KSHV orf10 failed to induce USP24 and p53 expression and phosphorylation in U2OS-ΔUSP24 cells compared to untransfected control (UN) (Fig 8D, compare lanes 1 and 2 to lanes 5 and 6). In the presence of etoposide, USP24 was no longer induced by KSHV ORF10, as it was deleted, and p53 levels were reduced in KSHV orf10 expressing as compared to untransfected control. This is most evident in the quantitation table below, which shows induction of p53 and Ser15-phosphorylated p53 relative to untransfected control in USP24 wild-type, but not USP24 deleted cells under both etoposide-induced and normal conditions. These data corroborate our hypothesis and further confirm USP24 role in KSHV orf10-dependent p53 induction.

## Discussion

A substantial number of viral proteins have no known function. An even greater number of hypothetical orfs are predicted for viral genomes. For these predicted orfs, it is not clear whether they are expressed during natural infection or can even be translated *in vitro*. The ORFEOME project was designed to bring a functional systems biology approach to address this knowledge gap and to test whether these uncharacterized orfs impinge on specific cellular signaling pathways (reviewed in [22]). This project set out to test the hypothesis that all viruses encode some proteins that interfere with p53 signaling, because p53 is a central node in cell danger response (CDR) signaling.

Numerous examples exist for viral proteins that interfere with p53 function, irrespective of virus family, genome organization and life style (latent or lytic, oncogenic, or acutely toxic) and regardless of the mode of viral replication (DNA or RNA) (reviewed in [2,5,12]). Not all known virus-p53 interactions are direct, but all affect p53 function; therefore, a functional screen, rather than an interaction screen, was employed.

This screen included 172 genes, including orfs predicted to encode proteins, known miRNAs, as well as suspected lncRNAs from eight diverse, emerging viruses. The screen identified two novel modulators of p53 signaling, KSHV orf10 and ZIKV NS2A. It confirmed the previously reported interaction between KSHV orf25 and p53 [32]. This screen also identified KSHV orf45 as affecting p53 signaling, which will be discussed elsewhere. By design, the screen did not include proteins with assigned biochemical functions even if those included p53 binding, such as KSHV orf73/LANA [45–47]. Key to the discovery was the use of a robust physiological inducer of p53 signaling, the DDR, and a cell line, U2OS that harbors fully functional, wild type p53. U2OS cells have been used for numerous studies and found to possess wild type p53 signaling while having a high transfection efficiency. The screen used a tumor-derived dominant negative mutant of p53 as positive control that has a well understood mechanism of action, based exclusively on heterodimer formation in the nucleus. Thus, inhibition by p53-273 is downstream of DDR induced activation (etoposide), and downstream of Nutlin-3 induced release of p53 from HDM2. This established a biological context in which to investigate p53 signaling. Conversely, the initial screen was validated in U87MG and tertHUVEC cells, two cell lines that are known to recapitulate central aspects of the biology for the two viruses ZIKV and KSHV, as the hits from the primary screen were proteins encoded by ZIKV and KSHV.

ZIKV is a positive-sense (+) single-stranded (ss) RNA virus that belongs to the family of Flaviviridae. ZIKV is associated with congenital neurological disorders and microcephaly due to its ability to cross the placenta barrier and target cortical neural progenitor cells (reviewed in [48,49]). Several studies have reported that ZIKV infection induces DDR, p53 activation, and apoptosis in neural progenitor cells [50,51]. Some studies have implicated the ZIKV structural and envelope proteins C, M, and E in the induction of p53 [52–54]. Another study only predicted NS5 as an interaction partner of p53, but not C, M, or E; this study did not include NS2A [55]. Capsid proteins (C) encoded by ZIKV and West Nile Virus (WNV) have been shown to bind to HDM2 and interfere with formation of the HDM2:p53 complex, which resulted in p53 stabilization [56]. In this screen, ZIKV C and ZIKV E did not have reproducible effects on p53-dependent transactivation, perhaps due to cell type specific modulation of protein function. As uncontrolled p53 induction leads to rapid apoptosis, one would expect that in addition to proteins such as C, M and E, which activate p53, ZIKV would also evolve the means to inhibit p53 function. In the flavivirus hepatitis C virus (HCV), NS3/4A and NS2 proteins induce mislocalization of ATM and p53 to the cytosol, respectively [16,17]. No data exist as to the interactions of other flavivirus proteins with p53. This screen identified ZIKV NS2A as a flavivirus-encoded suppressor of p53 signaling. ZIKV NS2A encoded functions: (i) affected the accumulation and/or localization of the DDR sensing kinase ATM and (ii) interacted with p53 and sequestered it in the cytosol. Viewed from a very high level, it seems as if ZIKV NS2A encodes functions that replicate the HCV NS3/4A and NS2 activities.

Other functions of ZIKV NS2A have also been reported. ZIKV NS2A inhibits IFN-γ production [57] and degrades adherence junction proteins [58]. These findings are not mutually exclusive, as most viral non-structural proteins have multiple functions and binding partners. Different partners become relevant in different tissues, in different cellular contexts or at different times in the infectious cycle. The p53 protein has also been implicated in IFN signaling [2]. Hence, affecting p53 could contribute to curbing IFN induction by ZIKV.

ZIKV differs from other flaviviruses because of its predilection to infect and replicate in neural progenitor cells. Premature differentiation of ZIKV-infected neural progenitors was detected in studies in human brain organoids [59]. ZIKV NS2A, but not DENV NS2A, led to decreased proliferation and premature differentiation of radial glial cells and impaired adherence junction formation in these cells [58]. The fact that p53 has an established role in neural stem cell proliferation and differentiation [60–63] suggests that the NS2A:p53 interaction, as established in the current study, may potentially contribute to premature differentiation of radial glial cells as well. As an aside, p53 inactivation by NS2A has implications for studies that propose to use ZIKV as an oncolytic agent for glioblastoma [64–67], since 68% of glioblastoma tumors harbor wild-type p53 [68]. It may be scientifically rewarding to study ZIKV-p53 interactions in the context of cancer therapy as well as during natural infection.

KSHV, or human herpes virus 8, is a double stranded DNA virus associated with several malignancies such as Kaposi’s sarcoma, primary effusion lymphoma, and multicentric Castleman’s disease (reviewed in [69]). KSHV is known to activate the DDR during lytic replication [70] and p53 is wild-type in KSHV-associated lymphoma and in Kaposi Sarcoma [71]. This suggests that KSHV evolved one or more mechanisms to counteract p53 during the different phases of its life cycle. This screen identified the lytic KSHV orf10 protein as a novel positive modulator of p53. KSHV orf10 interacted with the deubiquitinase USP24 and with p53 itself, and p53 is a known substrate for USP24 [43]. Hence, it is plausible to think that KSHV orf10 is part of a trimolecular complex leading to reduced p53 ubiquitination and increased activity.

KSHV orf10 has other binding partners in addition to p53. The KSHV orf10 protein is detectable in both the nucleus and the cytoplasm. KSHV orf10 reportedly inhibits type I IFN signaling by forming a complex with IFN receptor subunits, the Janus kinases Jak1 and Tyk2, and STAT2 transcription factor [72]. Furthermore, KSHV orf10 interacts with nuclear pore proteins, including Nup98 [40]. P53 nuclear export is CRM1-dependent, and Nup98 is implicated in CRM1-dependent nuclear export of proteins [41,73]. Therefore, we initially speculated that KSHV orf10 might bind directly to CRM1 or that KSHV orf10 binding to Nup98 disrupts its interactions with CRM1. This turned out not to be the case. Considering that Nup98 is also important for nuclear export of p21 mRNA [74] adds another layer of complexity. KSHV orf10 modulating activity towards Nup98 may regulate p21 expression secondary to its interaction with p53; however, the primary effect of KSHV orf10 towards p53 seems to be the inhibition of ubiquitination. Since the ubiquitin modification system is essential, universal, and evolutionarily conserved, it is tempting to speculate that cellular deubiquitinases in general have an anti-viral role by detecting viral protein surfaces, analogous to the pro-viral role that ubiquitin ligases such as E6-AP have in the life cycle of human papilloma virus [75].

Depending on the context, p53 induces either apoptosis or cell cycle arrest. Many herpesviruses can replicate in cell cycle-arrested cells [76], by virtue of encoding an army of enzymes to synthesize nucleotides independently of cellular anabolism. In fact, it may be beneficial for the viral DNA-dependent DNA polymerase to eliminate competition from the host DNA polymerases. Consistent with this idea, early studies suggested that KSHV induces cell cycle arrest during lytic replication and that p53 and p21 were necessary for KSHV lytic replication [77–79]. KSHV proteins, other than KSHV orf10 interact with p53 [32,45]. These include proteins, such as LANA, that are expressed in the latent, quiet phase of the life cycle, where viral genome replication is in synchrony with the host cell cycle and dependent on host DNA polymerase. This suggests, again, that the cellular context, such as lytic versus latent in the case of herpesviruses, or early versus late in the replication cycles of other viruses, makes a difference as to which viral proteins interact with p53 and what the physiological consequences of such interactions are.

In this differentiated view of the role of p53 in herpesvirus replication, it is worth considering a recent report on human cytomegalovirus (HCMV) [80]. It showed that egress and secondary envelopment of HCMV virions was significantly reduced in p53 knock-out cells and that “adding back” p53 rescued the phenotype. P53 was critical for expression of the early replication protein UL44, the nuclear egress complex protein UL53, and the structural proteins pp28/UL99 and MCP/UL86 [81,82], and loss of p53 altered the sub-cellular localization of the major tegument protein pp65/UL83 and the nuclear egress complex protein UL50. Perhaps there exist similar requirements for p53 in KSHV capsid formation. KSHV orf10 would help in setting up a cellular environment that is permissive for KSHV DNA replication, assembly, packaging, and egress. Studies on the HCMV pUL29/28-pUL38-p53 protein complex provide another example on which to model the function of the KSHV orf10-p53-UPS24 complex. The HCMV pUL29/28 protein, but not HCMV pUL38, directly binds to p53, yet both proteins are important for p53 stabilization. HCMV pUL38, but not pUL29/28, is able to regulate p53 transcriptional activity, resulting in selective suppression of p53 target genes [83]. HCMV pUL38 directly binds to USP24, and these interactions are essential for HCMV pUL38-dependent inhibition of apoptosis [84]. This firmly established, if somewhat convoluted, mechanism may also apply to KSHV orf10-p53 interactions: there may exist additional KSHSV-encoded partner proteins of KSHV orf10 mimicking HCMV pUL38 functions and conferring specificity as to which p53 target genes are inhibited.

In summary, the ORFEOME project provides a resource to the scientific community by making available sequences and expression-verified clones for 172 uncharacterized, ill-characterized, hypothetical or predicted genes for eight different, newly emerging viruses. It extends the paradigm of systems biology to include functional high-throughput screens. It also reaffirms the role of p53 not just in cancer, but also in innate immune signaling through the identification of two novel p53-interacting proteins, each from a different virus: ZIKV NS2A and KSHV orf10. It seems likely that every virus has evolved strategies to regulate p53 function; the mechanism depends very much on the context of infection.

## Material and methods

### Cell lines

Human embryonic (HEK) 293 cells, U2OS human osteosarcoma cells, and A549 *human* adenocarcinoma *alveolar basal epithelial cells* were grown in Dulbecco's Modified Eagle Medium (DMEM, Gibco, Thermo Fisher) supplemented with 10% Fetal Bovine serum (FBS). U87-MG human glioblastoma cell line [85] and *Human Umbilical Vein Endothelial Cells* (HUVEC) were grown in Minimum Essential Medium (MEM) alpha (Gibco, Thermo Fisher) and Endothelial Growth Medium (EGM, Lonza)-2%FBS supplemented with EGM Bullet Kit components (Lonza). U2OS-Cas9 cells [86] were grown in DMEM-10%FBS-10 μg/ml blasticidin. HEK293T.219 cells carrying KSHV genome were generated by cell-free transmission of rKSHV.219. Kaposi’s sarcoma-associated herpesvirus (KSHV) was isolated from iSLK.219 cells harboring latent recombinant KSHV.219 (rKSHV.219).[87] iSLK.219 cells were maintained in DMEM supplemented with 10% tetracycline-free FBS, G418 (250 μg/ml), hygromycin (400 μg/ml), and puromycin (10 μg/ml). To produce infectious virions, the cells were stimulated with 3 μg/ml doxycycline, and 1 mM sodium butyrate [88]. After 72 h, supernatant was harvested, and cell debris was pelleted and filtered through a sterile 0.45-μm filter. To determine the infectious titer, Vero cells were treated with concentrated virus and the number of GFP-expressing cells 48 hours postinfection was counted. Dividing the number of GFP-expressing cells by the volume of concentrated virus used yielded infectious units (IU)/ml. To generate the 293T.219 cell line, 7.5 x 105 HEK 293T cells were seeded in a 6-well plate in DMEM supplemented with 10% FBS (Sigma). After 24 hours, the medium was changed to Opti-MEM I reduced-serum medium (Thermo Fisher), and the cells were treated with concentrated virus at a multiplicity of infection (MOI) of 1 in the presence of polybrene (10 μg/mL) and spin-inoculated at 2,500 rpm for 90 minutes at 30°C. Immediately following centrifugation, the medium was supplemented with 10% FBS, and the cells were incubated at 37°C for 16 hours before changing the medium to DMEM supplemented with 10% FBS. To select for stably infected cells, the medium was supplemented with puromycin (1 μg/mL) 48 hours post infection, and the cells were maintained in this selective medium henceforth.U2OS-ΔUSP24 cell line was generated using CRISPR/Cas9 as described elsewhere [86]. Briefly, U2OS-Cas9 cells were grown to 80% confluency and transduced with CRISPR gRNA lentiviral transduction particles (multiplicity of infection of 5.0, Sigma-Aldrich) carrying a USP24 specific sgRNA (sgRNA-1: 5’-GATGGCCGGTACACTTACC-3’, clone ID HSPD0000072983; sgRNA-2: 5’-TCGAGCCACTTAAGCCTCC-3’, clone ID HSPD0000072984; sgRNA-3: 5’-AAGTTTGATCGCTTGTCAT-3’, clone ID HSPD0000072986) using 10μg/ml polybrene and spinfection (1000xg, 90 min). Transduced cells were selected with 10mg/ml blasticidin and 5 μg/ml puromycin. Deletion of USP24 was confirmed by PCR (forward primer 5’-ACACAAGGCCTGATACCTCC-3’ and reverse primer 5’-ACTGCAAATTCTCATTACCACTTT-3’) and immunoblotting with anti-USP24 Ab.

### Plasmid constructs

PG13-luc (pGL13), a firefly luciferase reporter plasmid containing thirteen p53 binding elements; and pCMV-Neo-Bam and vectors expressing wild-type and dominant-negative mutant p53 pCMV-Neo-Bam-p53WT and pCMV-Neo-Bam- *p53*R273H (pC53-*4*.*2N3)*, respectively, were described elsewhere [89,90]. pGL3-Basic control vector expressing firefly luciferase under SV40 promoter was purchased from Promega. Plasmid constructs expressing orfs encoded by Zika virus (ZIKV), Chikungunya Virus (CHIKV), Ebola, Influenza A, Severe acute respiratory syndrome (*SARS*), Middle East Respiratory Syndrome (MERS) coronavirus (MERS-CoV), bat CoV, Kaposi Sarcoma Associated Herpesvirus (KSHV) (S1 Table; https://www.med.unc.edu/orfeome) were cloned using gateway cloning system (Invitrogen). A subset of orf constructs were synthesized by Bio Basic, Amherst New York. NLS-mCherry-NES reporter vector (pDN160) was a gift from Barbara Di Ventura & Roland Eils (Addgene plasmid # 72660; http://n2t.net/addgene:72660; RRID:Addgene_72660 [91]). pOME0304, pOME0004, pOME0184, referred to as pDEST47-ZIKV NS2A-Flag, pDEST47-KSHV orf10-Flag, and pDEST47-KSHV orf57-Flag, respectively, express proteins fused to the C-terminal 3xFlag epitope (S1 Table). A vectors expressing KSHV orf45 and KSHV orf25 tagged with C-terminal Strep-tag, referred to as ‘KSHV orf45g’ and ‘KSHV orf25g’, respectively, were described elsewhere [42]. All quality control data, plasmid maps, and sequences are available through the ORFEOME website. Plasmid sequences are also available through VIPR.

### Drugs and antibodies

Etoposide (MP Biomedicals, 02193918-CF), nutlin-3 (Millipore Sigma, N6287), MG132 (Cayman Chemicals, 10012628), N-Ethylmaleimide (NEM, Millipore Sigma, E3876), leptomycin B (LMB, Millipore Sigma, L2913), mouse anti-Flag (M2, Millipore Sigma, F1804), rabbit anti -Flag (D6W5B, Cell Signaling, 14793), mouse anti -p53 pAb421 (Novus Biologicals, NBP2-62555), mouse anti -p53 (DO-7, Invitrogen, MA5-12557), rabbit anti -p53 pSer15 (Cell Signaling, 9284), mouse anti -ATM (11G12, Cell Signaling, 92356), rabbit anti -ATM pSer1981 (D25E5, Cell Signaling, 13050), mouse anti -ATR (2B5, GeneTex, GTX70109), rabbit anti -ATR pSer428 (Cell Signaling, 2853), mouse anti -p21 (187, Santa-Cruz, sc-817), rabbit anti -H2AX (Cell Signaling, 2595), rabbit anti -H2AX pSer139 (20E3, Cell Signaling, 9718), mouse anti -actin (Abcam, ab8226), rabbit anti-USP24 (Abcam, ab72241), rabbit anti -ubiquitin (Cell Signaling, 3933), rabbit anti -NUP98 (C39A3, Cell Signaling, 2598), rabbit anti -Exportin-1/CRM1 (D6V7N, Cell Signaling, 46249), mouse TrueBlot (Rockland, 18-8817-33), horse anti -mouse horse reddish peroxidase (HRP) conjugated (Vector Laboratories, PI-2000), goat anti-rabbit HRP (Vector Laboratories, PI-1000), horse anti -mouse conjugated to fluorescein isothiocyanate (FITC, Vector Laboratories, FI-2000), goat anti-rabbit Texas Red (Vector Laboratories, TI-1000), VectaFluor Excel Dylight 488 anti-mouse IgG (Vector Laboratories, DK-2488), VectaFluor Excel Dylight 594 anti-rabbit IgG (Vector Laboratories, DK-1594), p53-N-term-Trap-A (epitope AA 1–8, ChromoTek, pta-10), p53-C-term-Trap-A (epitope AA 302–393, ChromoTek, pta2-10), Binding control (ChromoTek, bab-20), Protein A/G Plus-agarose (Santa Cruz, sc-2003).

### P53-Luc assay

For ‘plasmid P53-overexpression’, HEK293 cells were co-transfected with pGL13, pCMV-Neo-Bam-p53WT and ether pCMV-Neo-Bam, pCMV-Neo-Bam*-p53*R273H, or a vector expressing an orf of interest using Lipofectamine 2000 (Invitrogen, 11668027) according to manufacturer’s protocol. At 24h post transfection (p.t.), luciferase expression levels were measured using ONE-Glo luciferase assay system (Promega, E6110). For induction of endogenous p53 with etoposide or nutlin-3, U2OS cells were transfected with pGL13 and either pCMV-Neo-Bam, pCMV-Neo-Bam-*p53*R273H, or a vector expressing an orf of interest using Lipofectamine 2000. At 18h p.t., the cells were overlaid with fresh medium containing 5 μM etoposide or 10 μM of nutlin-3 and incubated for additional 24h before measuring luciferase expression with ONE-Glo luciferase assay system. Cell viability to determine cytotoxicity of each expressed orf or the drug treatment was measured with CellTiter-Glo luminescent cell viability assay (Promega, G7570).

### Immunoprecipitation

U2OS cells were transfected with a vector expressing an orf of interest using Polyethylenimine (PEI, Linear, MW 25000, Polysciences, Inc., 23966–1) according to a previously described protocol with minor modifications [92]. DNA PEI solution was prepared at 1:3 ratio in Opti-MEM (Gibco, 31985062) and incubated for 10 min at room temperature (RT) before adding to the cells. At 18h p.i, the cells were either stimulated with 10μM etoposide or left untreated and then incubated for additional 24h. Harvested cells were lysed in 50mM Tris (pH7.4)-5mM ethylenediaminetetraacetic acid (EDTA)-0.5% Nonidet P-40 (NP-40)-150mM NaCl-5% sucrose (TENN) buffer supplemented with cOmplete protease inhibitor cocktail (Millipore Sigma, 11697498001) for 30 min on ice with occasional vortexing and spun down at 15,000xg for 10 min. The lysates were pre-cleared by incubation with protein A/G plus agarose beads (Santa-Cruz, sc-2003) or ‘binding control’, non-conjugated agarose beads (ChromoTek, bab-20) and co-immunoprecipitated with either mouse anti-Flag antibody and protein A/G plus agarose beads or *p53-Trap N*-term_A and *p53-Trap* C-term_A (ChromoTek, pta-10 and pta2-10), agarose-conjugated proteins specific for p53 N- or C-terminus, respectively. The beads were washed with 50mM Tris (pH7.4)-5mM EDTA-1% or 0.5% NP-40-500mM NaCl (SNNTE) buffer. The samples were analyzed by immunoblotting with indicated Ab.

### p53 Ubiquitination assay

U2OS cells transfected with a vector expressing an orf of interest using PEI as described above were treated with 30μM MG132 for 6h and then incubated with 10mM NEM for 10 min before harvest. Samples were lysed in radioimmunoprecitation (RIPA) buffer (50mM Tris (pH7.4)-150mM NaCl-1% Triton X-100-0.1% *Sodium dodecyl sulfate* (SDS)-1% *Sodium deoxycholate)* supplemented with 10mM NEM, Benzonase nuclease (Millipore Sigma, E1014), and complete protease inhibitor cocktail (Millipore Sigma, 1169748001) on ice for 30 min. The lysates were centrifuged at 15000xg for 10min at 4°C, pre-cleared with protein A/G plus agarose beads for 30 min at 4°C, and immunoprecipitated by incubation with anti-p53 pAb421 (18h, 4°C) and protein A/G plus agarose beads (1h, 4°C). The samples were washed with RIPA buffer and analyzed by immunoblotting with anti-ubiquitin Ab and anti-p53 Ab clone DO7.

### Immunoblotting

For protein accumulation assay (Fig 2), cells were lysed in RIPA buffer supplemented with Benzonase nuclease, and complete protease inhibitor cocktail. Otherwise, the samples were prepared as indicated. The samples were separated on 6% or 12% SDS- polyacrylamide gel electrophoresis (SDS-PAGE), transferred to Polyvinylidene fluoride (PVDF) membrane, blocked with 10% skim milk, and blotted with indicated Ab diluted either in 5% skim milk or 5% BSA.

### Immunofluorescence

The cells were plated onto coverslips and transfected with an expression vector. U2OS cells were transfected with PEI as described above. U87-MG and A549 cells and HUVEC cells were transfected with Lipofectamine LTX (Invitrogen, 15338100) according to the manufacturer’s protocol. At 18h p.t., the cells were stimulated with 10 μM etoposide for 1.5-2h and fixed/ permeabilized with ice-cold 100% methanol. Flag-conjugated proteins were stained with either mouse or rabbit anti-Flag Ab and goat anti -mouse or goat anti -rabbit Ab conjugated to TexasRed or fluorescein isothiocyanate (FITC). Endogenously expressed cellular proteins were stained with one of the following Ab (mouse anti -p53 DO7, rabbit anti -p53 pSer15, mouse anti -p21, rabbit anti -USP24, mouse anti -ATM, rabbit anti -ATM pSer1981, anti -ATR, and anti -ATR pSer428) and either VectaFluor Excel Amplified DyLight 594 Anti-Rabbit IgG Kit or VectaFluor *Excel Amplified Dylight* 488 anti-Rabbit IgG Kit (Vector Laboratories). The nuclei were stained with 4′,6-diamidino-2-phenylindole (DAPI). Z-stack images collected using LEICA DM4000B epi-fluorescence microscope by setting the lowest and highest plane of Z-axes were subjected to deconvolution using SimplePCI_5 version 6.2 microscope software. The individual optical sections were exported to multipage TIFF format and processed using Imaris version 7.1.0 software (Bitplane).

### Statistical analysis

All analyses were conducted using the R statistical language (version 3.5.3). The response curve of the screen was tested for “Normality” by QQ-plot and Shapiro–Wilk test and the contribution of individual orfs identified by general linear model. Dunnett's test was used to adjust for multiple comparison relative to “vector” and significance level alpha was set at p≤0.05.

## Supporting information

S1 Fig p53-Luc screening. The raw data are shown as a histogram (left panels) and the empirical cumulative distribution function (ECDF, right panels) after transfection of the ORFEOME expression plasmids for viability in HEK293 cells (A), p53 response as measured by p53 reporter after etoposide treatment in U2OS cells (B) and non-specific response as measured by Luc reporter expressed under a SV40 minimal promoter (C). For the histogram (left panels), frequency and log10 (RLU) are shown on the vertical on the horizontal axes, respectively. For the EDCF plot (right panels), the vertical axis is the ECDF, which represents the percentage of ORFEOME plasmids that yielded an output of less than or equal to a particular value of RLU shown on log10-scaled horizontal axis. The data were not scaled, nor batch adjusted.
(TIF)

S2 Fig Subcellular localization in U87-MG and HUVEC cells. U87-MG and HUVEC cells were transfected with pDEST47-ZIKV NS2A-Flag and pDEST47-KSHV orf10-Flag, respectively. After 18h-incubation, the cells were stimulated with 10 μM etoposide for 1.5h (A, C, E) or left untreated (B, D, F). The cells were fixed with methanol and stained with indicated Abs. Each image represents an individual optical section. The scale bar is 50 μM. The arrows point at the cells expressing either ZIKV NS2A or KSHV orf10.
(TIF)

S3 Fig ATR, and ATM expression in the presence of ZIKV NS2A. (A) U2OS cells were transfected with pCMV-Neo-Bam (EV), pDEST47-ZIKV NS2A-Flag, or left untransfected (UN). After 18h-incubation, the cells were stimulated with 10μM etoposide for 6h. The cell lysates were analyzed by SDS-PAGE and immunoblotting with indicated Abs. (B) U2OS cells, transfected with pDEST47-ZIKV NS2A-Flag. After 18h-incubation, the cells were stimulated with 10μM etoposide for 1.5h (B, D) or left untreated (C), fixed with methanol and stained with indicated Abs. Each image represents an individual optical section. The scale bar is 50μM. The arrows point at the cells expressing ZIKV NS2A.
(TIF)

S4 Fig KSHV orf10 does not induce DDR and does not interfere with CRM1-dependent nuclear export of proteins. (A) U2OS cells were transfected with pCMV-Neo-Bam (EV), pDEST47-KSHV orf10-Flag, or pDEST47-KSHV orf57-Flag and incubated for 24h or 48h. The cell lysates were analyzed by SDS-PAGE and immunoblotting with indicated Abs. (B) KSHV orf10 does not coimmunoprecipitate with CRM1. U2OS cells, transfected with pDEST47-KSHV orf10-Flag or left untransfected (UN) for 18h. orf10-Flag was immunopreciptated with mouse anti-Flag Ab. Presence of CRM1 or Nup98 in the lysates and coimunoprecipitated fractions was tested with protein-specific Ab. (C) KSHV orf10 does not interfere with CRM1-dependent nuclear export of NLS-mCherry-NES reporter protein. U2OS cells were transfected with expression plasmid for NLS-mCherry-NES alone (C, D) or together with KSHV orf10-Flag expressing plasmid (E) for 24h. As a control, cells transfected with NLS-mCherry-NES alone were incubated in the presence or absence of 10ng/μl leptomycin B (LMB) for 30min (D). The samples were fixed with methanol and stained with anti-Flag Ab to visualize KSHV orf10 expression. Each image represents an individual optical section. The scale bar is 50 μM.
(TIF)

S5 Fig Non-specific antibody reactivity for NS2A. (A) Amino acid sequence of ZIKA NS2A. Green indicated the predicted transmembrane segments. Also shown are the two peptides that were used to raise NS2A-specific antisera. (B) Specificity validation of the antisera, which unfortunately failed by Western-Blot analysis.
(TIF)

S6 Fig Originals of all Western-blots. (PDF)

S1 Table Plasmid constructs expressing viral orfs screened in p53-Luc assays. Plasmid constructs expressing orfs encoded by ZIKV, CHIKV, EBOV, IFA, *SARS*, MERS-CoV, bat CoV, and KSHV were cloned using the gateway cloning system and were validated by sequencing, immunoblotting, and RT-PCR. Additional and updated information is available at the web-site that accompanies this manuscript: (https://www.med.unc.edu/orfeome).
(PDF)
