# Supplementary figures and images for: Novel modulators of p53-signaling encoded by unknown genes of emerging viruses
Source: PLoS Pathog. 2021 Jan 7;17(1):e1009033. doi: 10.1371/journal.ppat.1009033 (PMC7790267; doi:10.1371/journal.ppat.1009033)

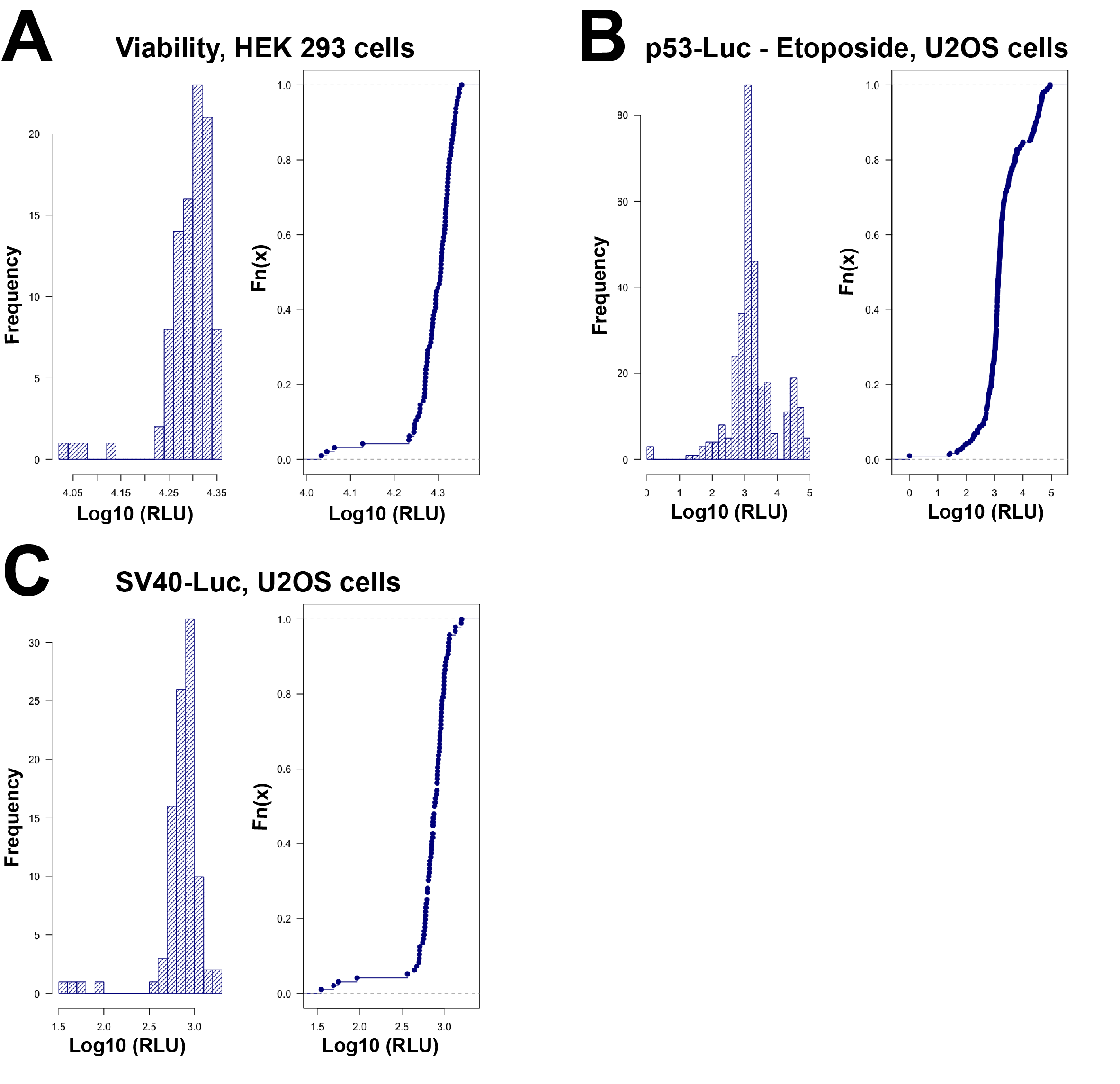

Supplement: S1 Fig — The raw data are shown as a histogram (left panels) and the empirical cumulative distribution function (ECDF, right panels) after transfection of the ORFEOME expression plasmids for viability in HEK293 cells (A), p53 response as measured by p53 reporter after etoposide treatment in U2OS cells (B) and non-specific response as measured by Luc reporter expressed under a SV40 minimal promoter (C). For the histogram (left panels), frequency and log10 (RLU) are shown on the vertical on the horizontal axes, respectively. For the EDCF plot (right panels), the vertical axis is the ECDF, which represents the percentage of ORFEOME plasmids that yielded an output of less than or equal to a particular value of RLU shown on log10-scaled horizontal axis. The data were not scaled, nor batch adjusted. (TIF) [file ppat.1009033.s001.tif]

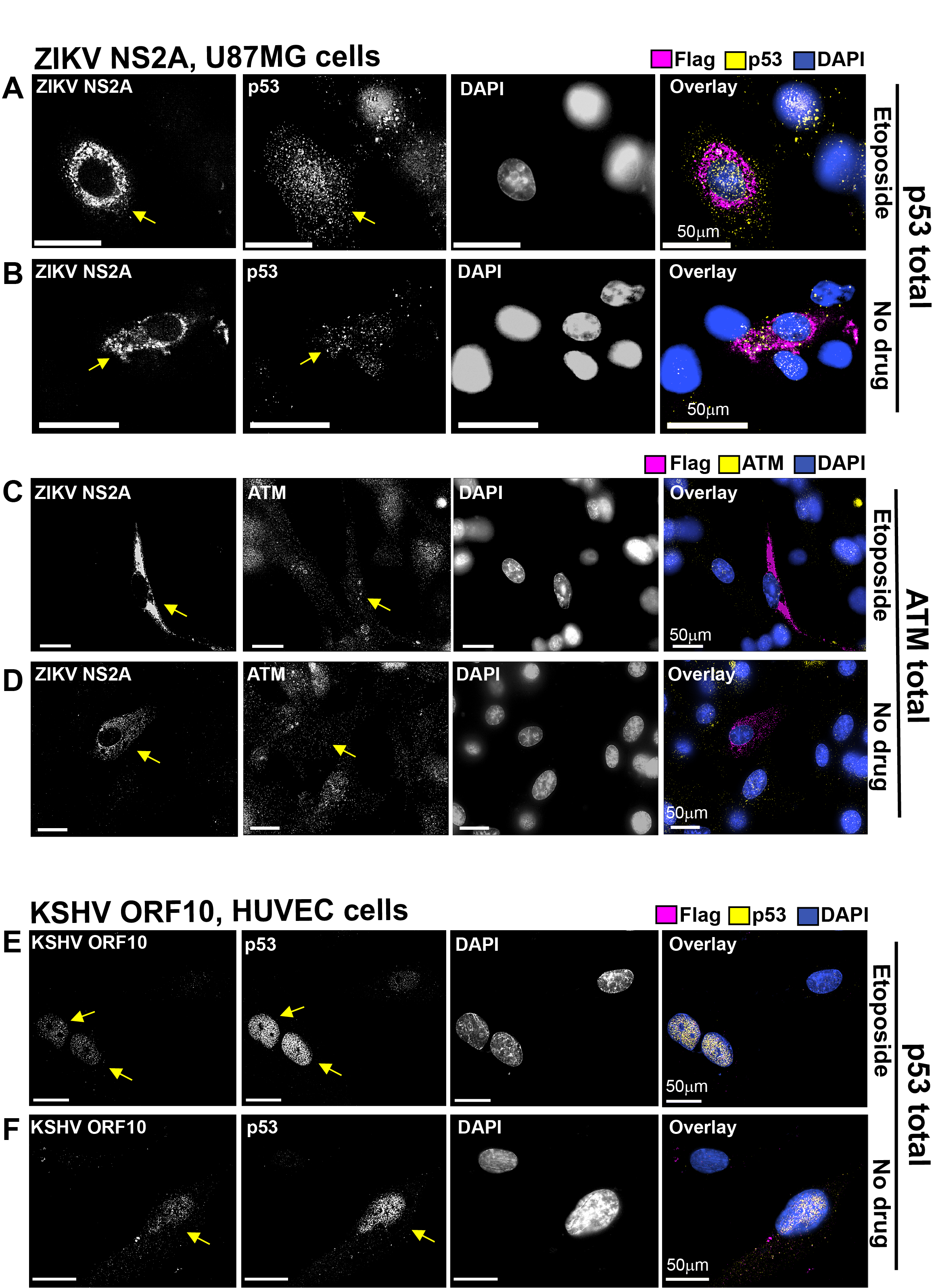

Supplement: S2 Fig — U87-MG and HUVEC cells were transfected with pDEST47-ZIKV NS2A-Flag and pDEST47-KSHV orf10-Flag, respectively. After 18h-incubation, the cells were stimulated with 10 μM etoposide for 1.5h (A, C, E) or left untreated (B, D, F). The cells were fixed with methanol and stained with indicated Abs. Each image represents an individual optical section. The scale bar is 50 μM. The arrows point at the cells expressing either ZIKV NS2A or KSHV orf10. (TIF) [file ppat.1009033.s002.tif]

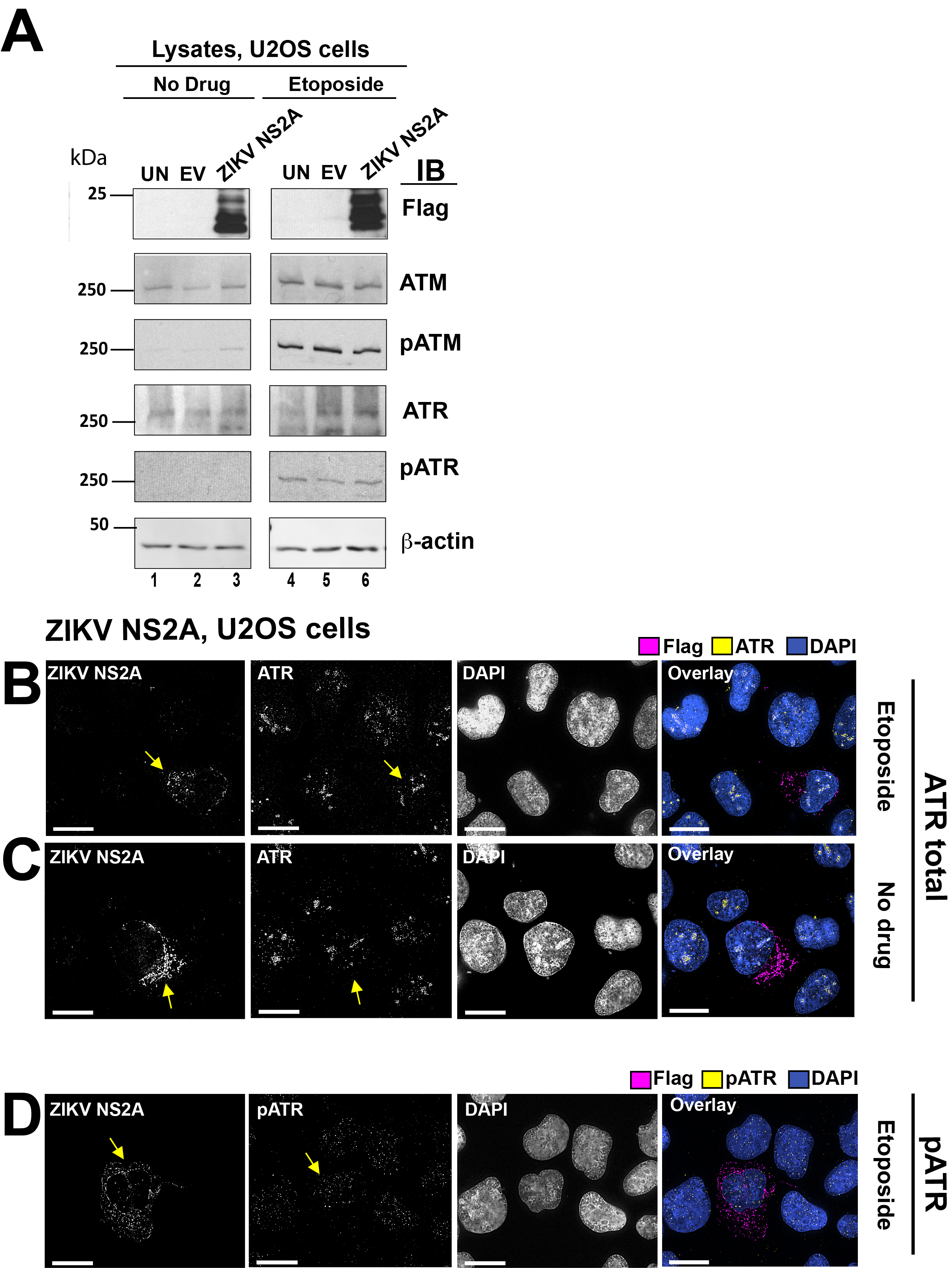

Supplement: S3 Fig — (A) U2OS cells were transfected with pCMV-Neo-Bam (EV), pDEST47-ZIKV NS2A-Flag, or left untransfected (UN). After 18h-incubation, the cells were stimulated with 10μM etoposide for 6h. The cell lysates were analyzed by SDS-PAGE and immunoblotting with indicated Abs. (B) U2OS cells, transfected with pDEST47-ZIKV NS2A-Flag. After 18h-incubation, the cells were stimulated with 10μM etoposide for 1.5h (B, D) or left untreated (C), fixed with methanol and stained with indicated Abs. Each image represents an individual optical section. The scale bar is 50μM. The arrows point at the cells expressing ZIKV NS2A. (TIF) [file ppat.1009033.s003.tif]

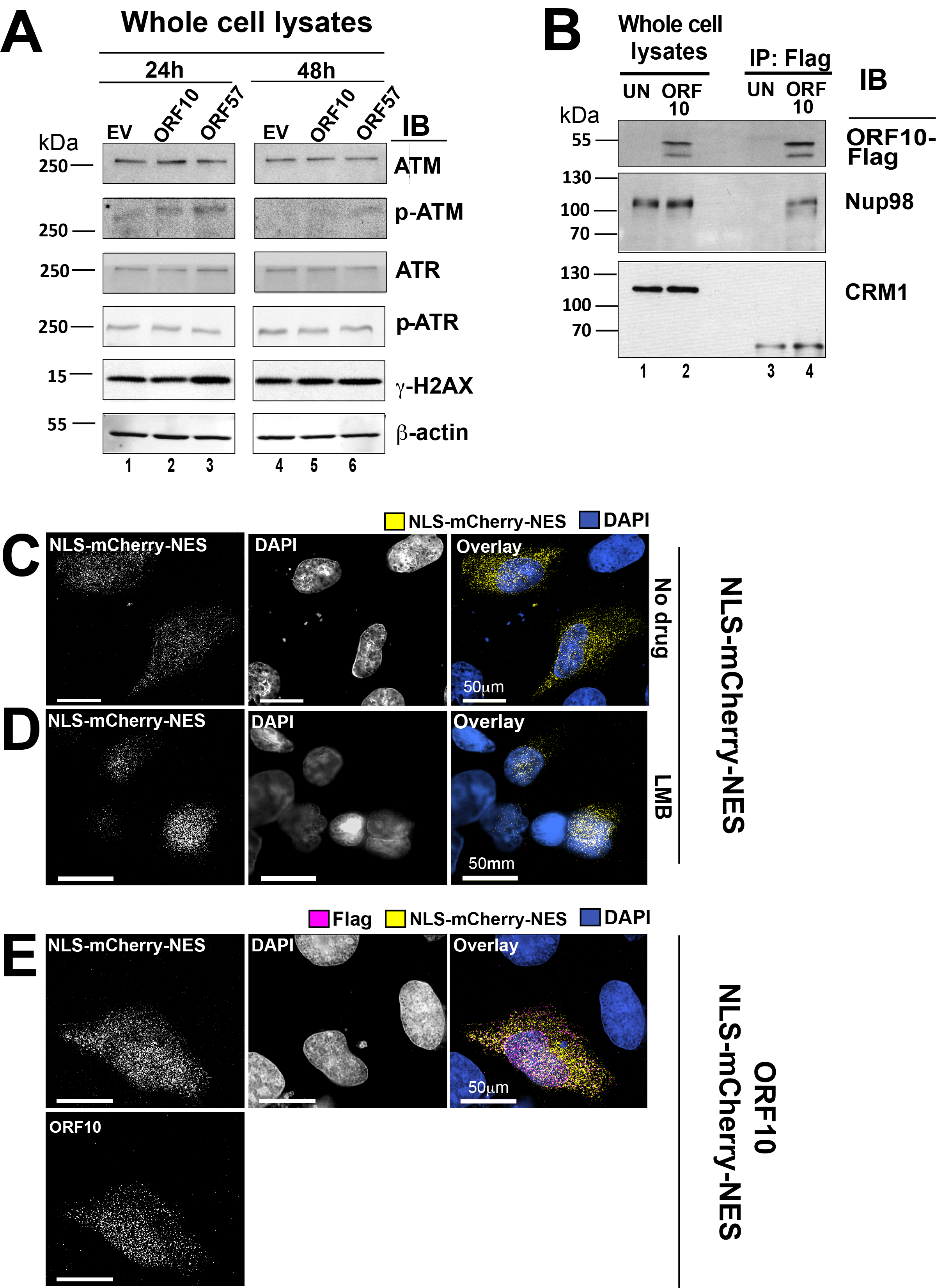

Supplement: S4 Fig — (A) U2OS cells were transfected with pCMV-Neo-Bam (EV), pDEST47-KSHV orf10-Flag, or pDEST47-KSHV orf57-Flag and incubated for 24h or 48h. The cell lysates were analyzed by SDS-PAGE and immunoblotting with indicated Abs. (B) KSHV orf10 does not coimmunoprecipitate with CRM1. U2OS cells, transfected with pDEST47-KSHV orf10-Flag or left untransfected (UN) for 18h. orf10-Flag was immunopreciptated with mouse anti-Flag Ab. Presence of CRM1 or Nup98 in the lysates and coimunoprecipitated fractions was tested with protein-specific Ab. (C) KSHV orf10 does not interfere with CRM1-dependent nuclear export of NLS-mCherry-NES reporter protein. U2OS cells were transfected with expression plasmid for NLS-mCherry-NES alone (C, D) or together with KSHV orf10-Flag expressing plasmid (E) for 24h. As a control, cells transfected with NLS-mCherry-NES alone were incubated in the presence or absence of 10ng/μl leptomycin B (LMB) for 30min (D). The samples were fixed with methanol and stained with anti-Flag Ab to visualize KSHV orf10 expression. Each image represents an individual optical section. The scale bar is 50 μM. (TIF) [file ppat.1009033.s004.tif]

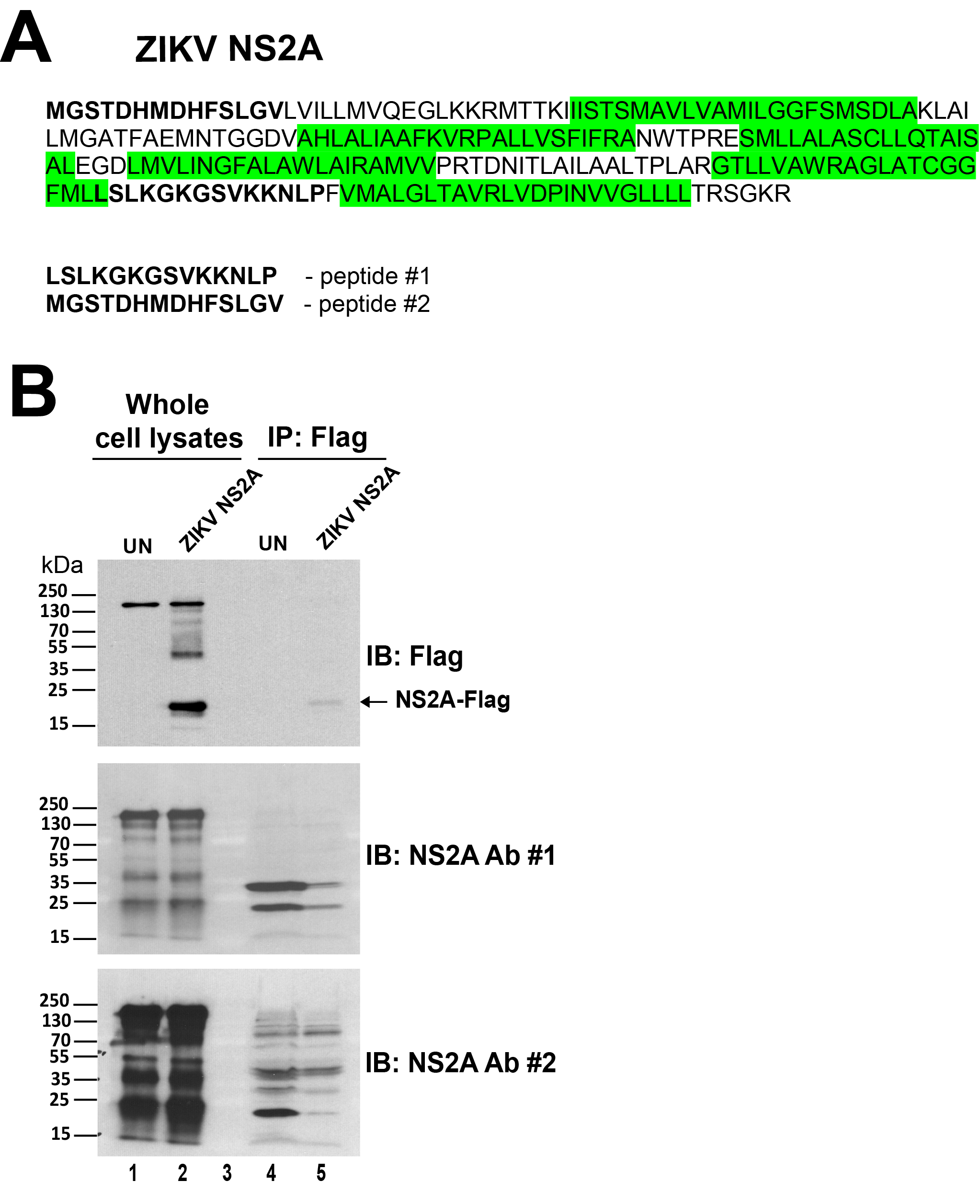

Supplement: S5 Fig — (A) Amino acid sequence of ZIKA NS2A. Green indicated the predicted transmembrane segments. Also shown are the two peptides that were used to raise NS2A-specific antisera. (B) Specificity validation of the antisera, which unfortunately failed by Western-Blot analysis. (TIF) [file ppat.1009033.s005.tif]
