# Supplementary material for: Novel modulators of p53-signaling encoded by unknown genes of emerging viruses
Source: PLoS Pathog. 2021 Jan 7;17(1):e1009033. doi: 10.1371/journal.ppat.1009033 (PMC7790267; doi:10.1371/journal.ppat.1009033)

A Original images for Figure 2A

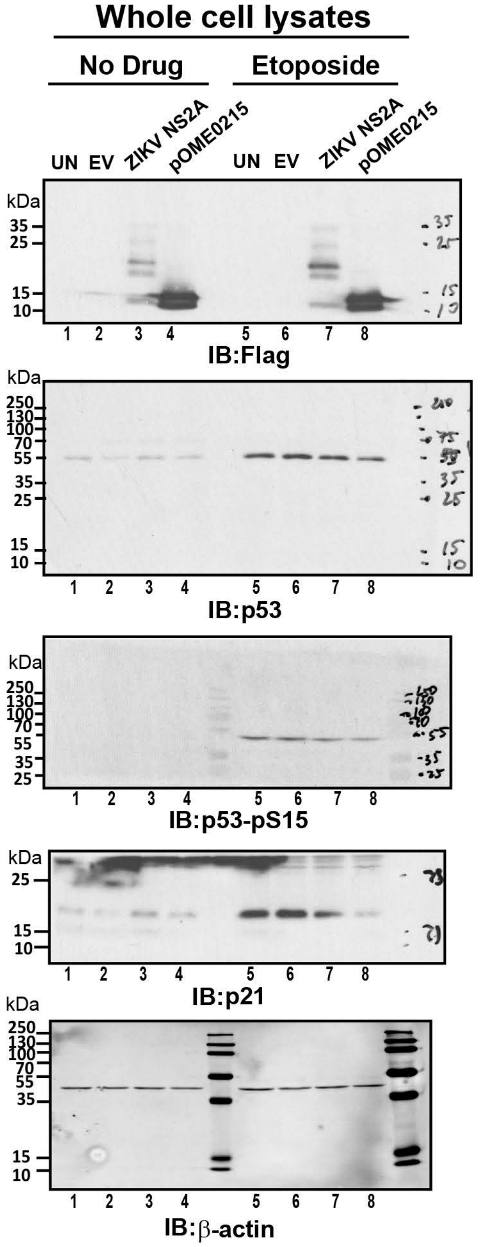

B Original images for Figure 3C

ZIKV NS2A-Flag pull-down

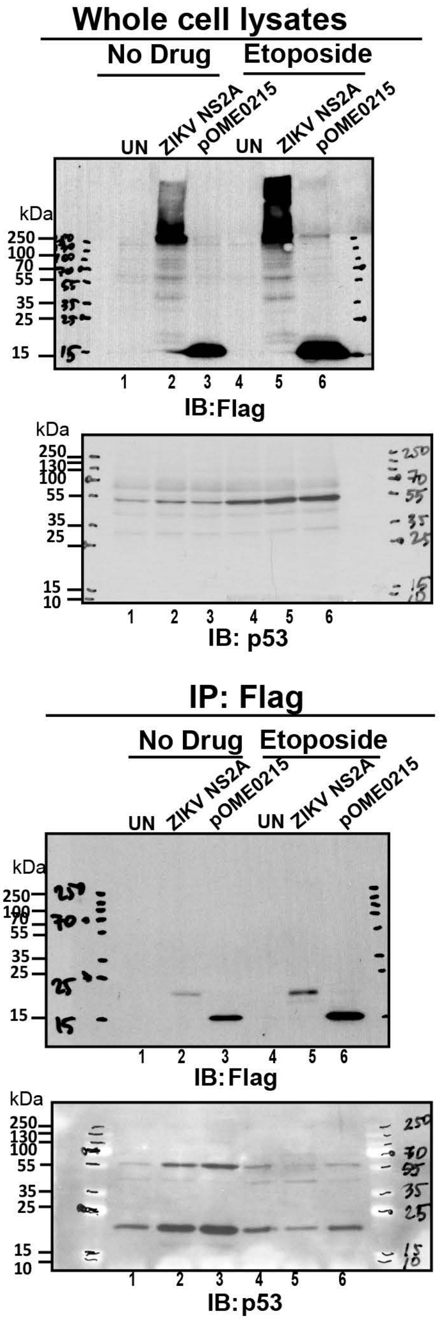

C Original images for Figure 3D

p53 pull-down

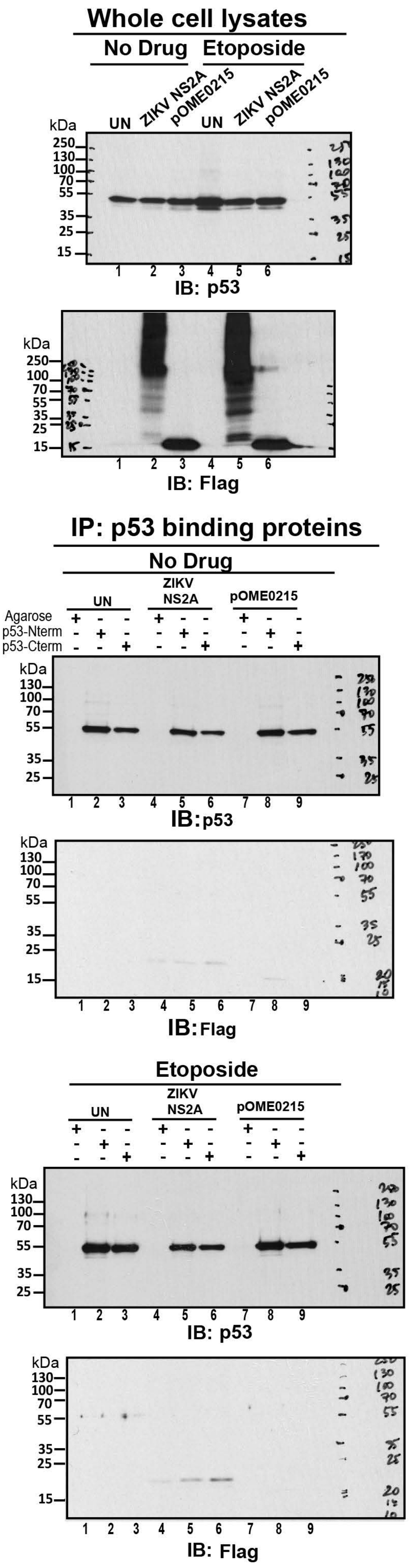

D Original images for Figure 5A

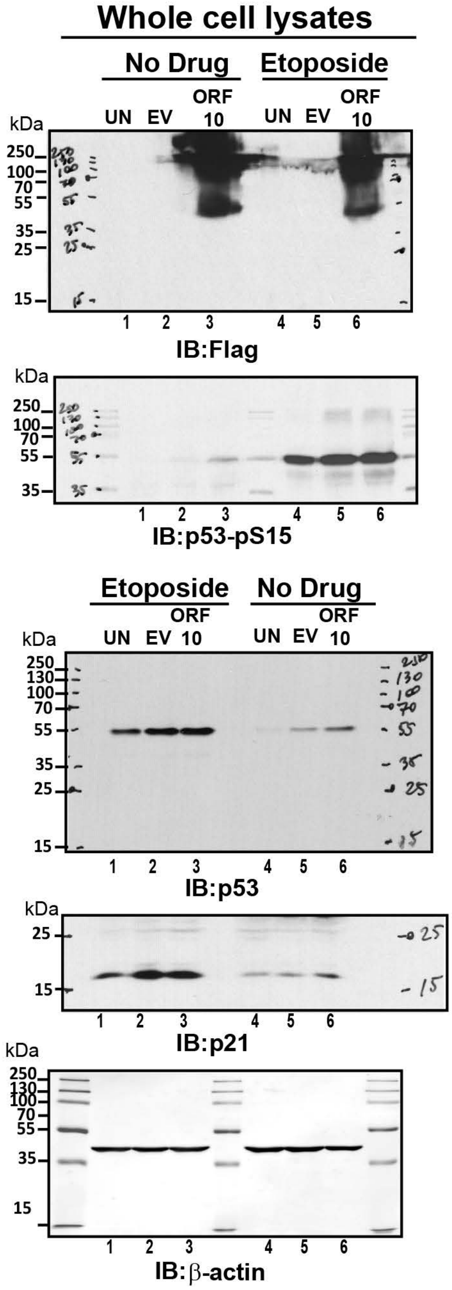

B Original images for Figure 6D

KSHV ORF10-Flag and USP24

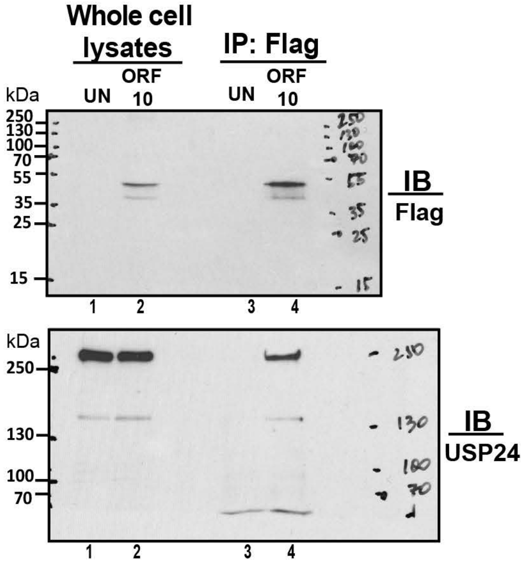

E Original images for Figure 6E

KSHV ORF10-Flag and p53

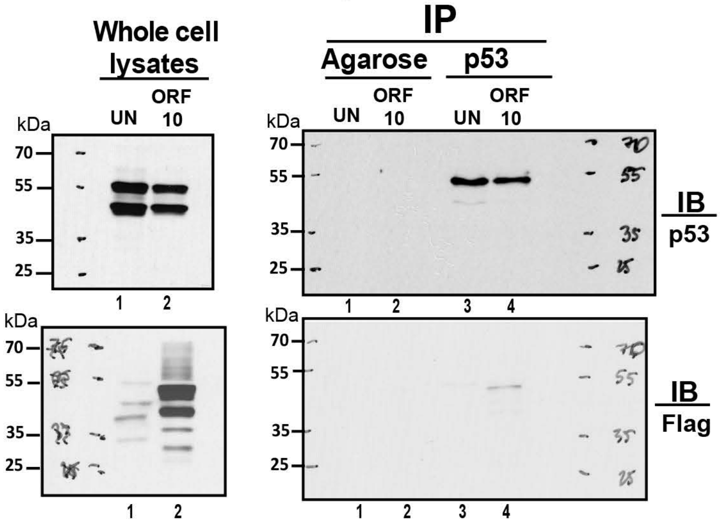

F Original images for Figure 6F

KSHV ORF10-Flag: p53 ubiquitination assay

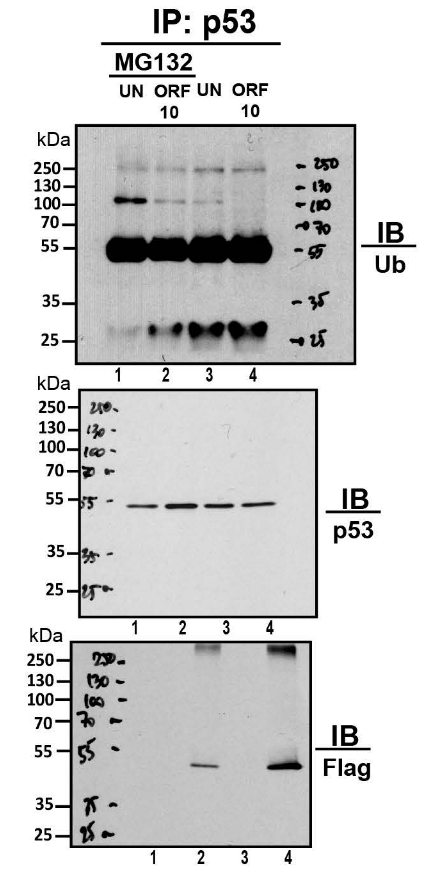

H Original images for Figure 7G

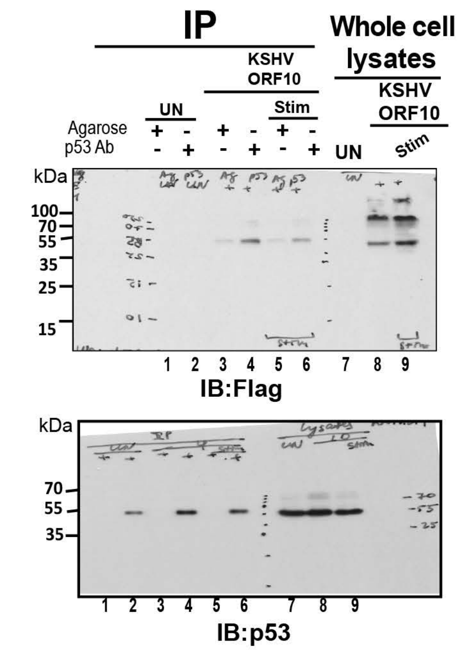

J Original images for Figure 8B

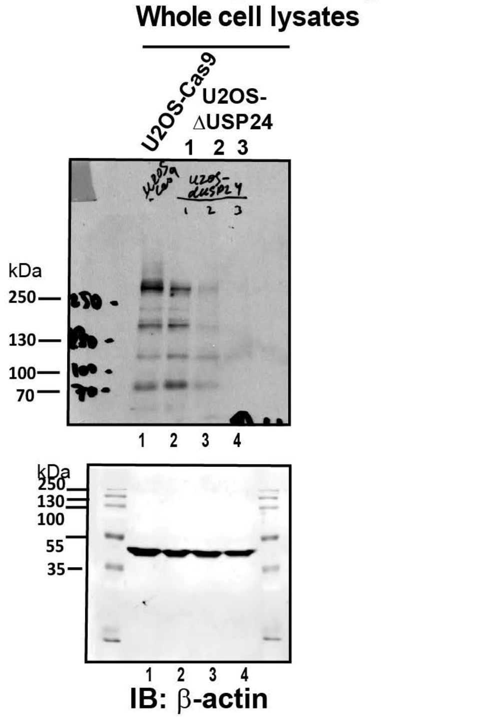

L Original images for Figure 8D

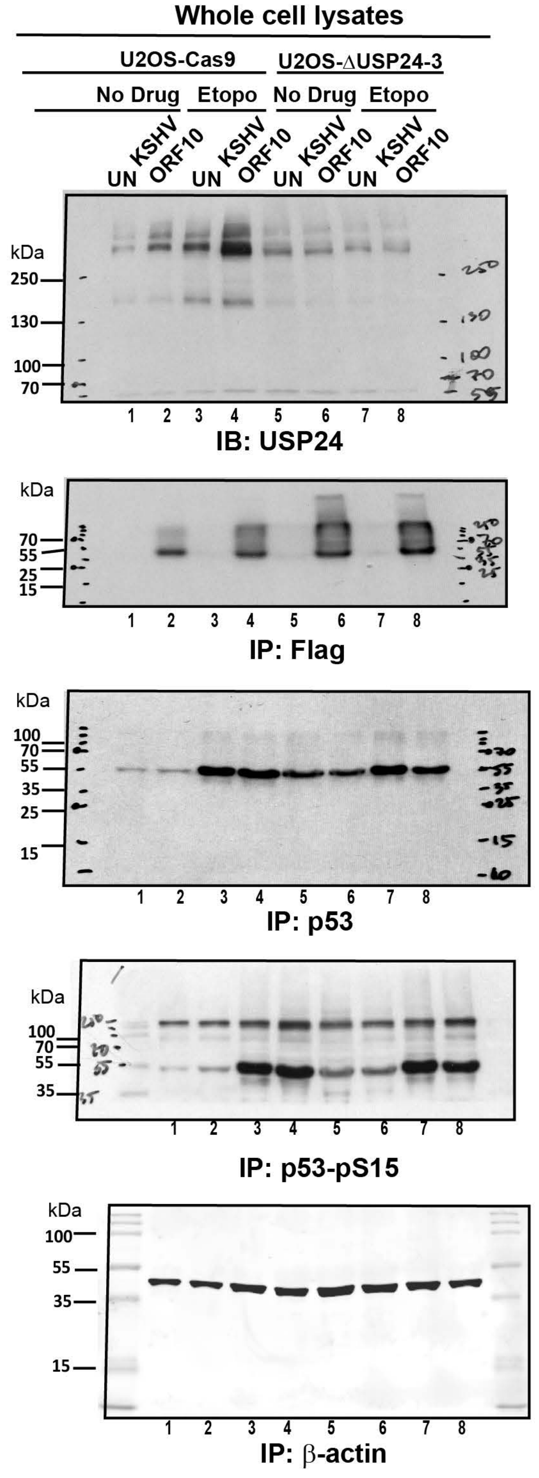

I Original images for Figure 8A

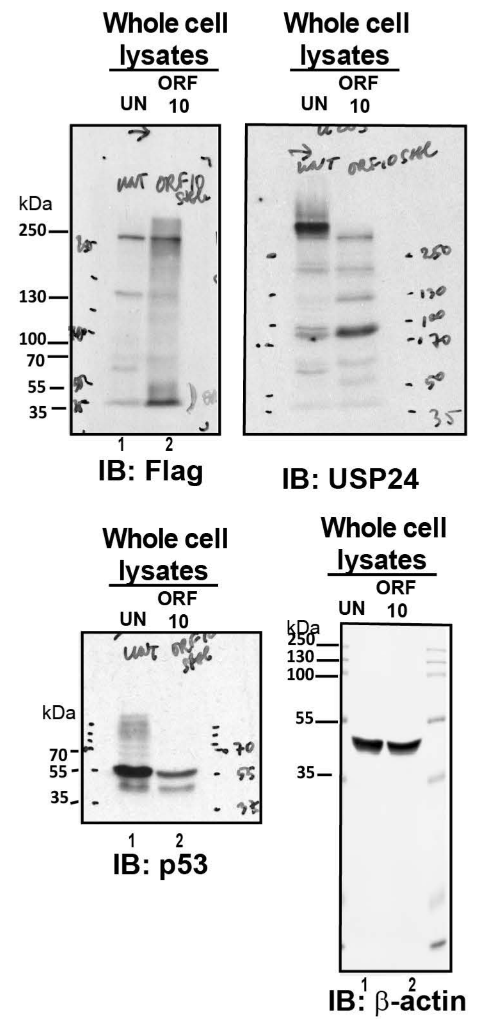

K Original images for Figure 8C

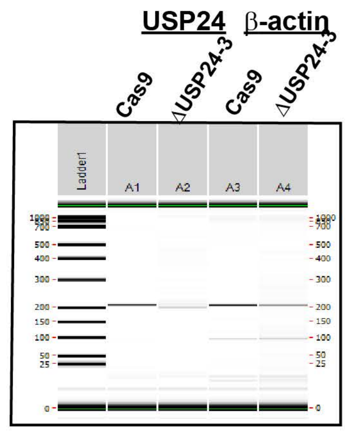

M Original images for Figure S3A

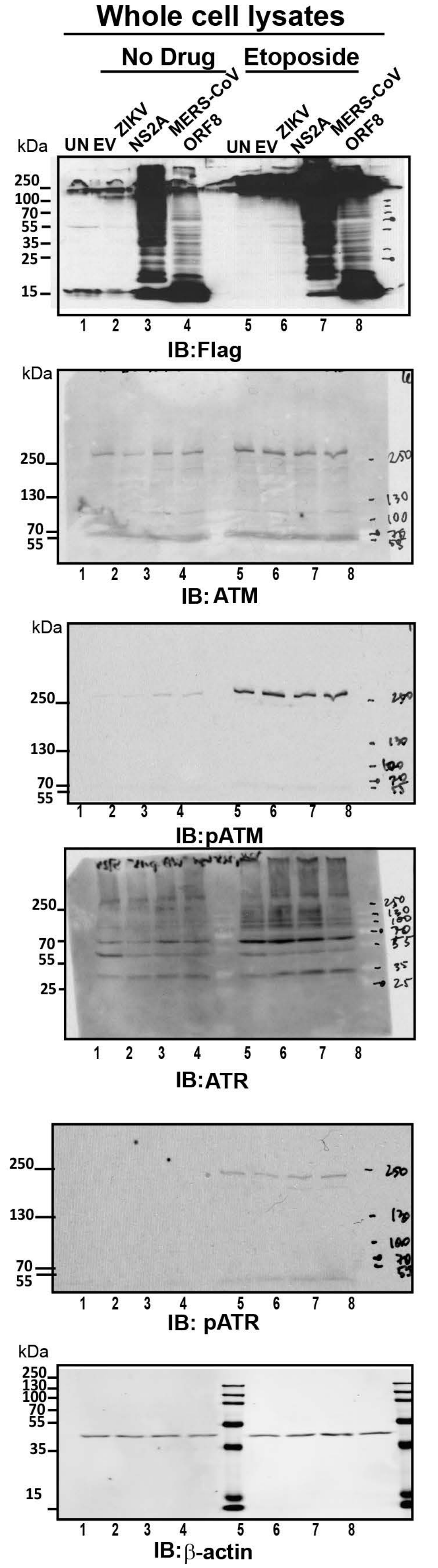

N Original images for Figure S4A

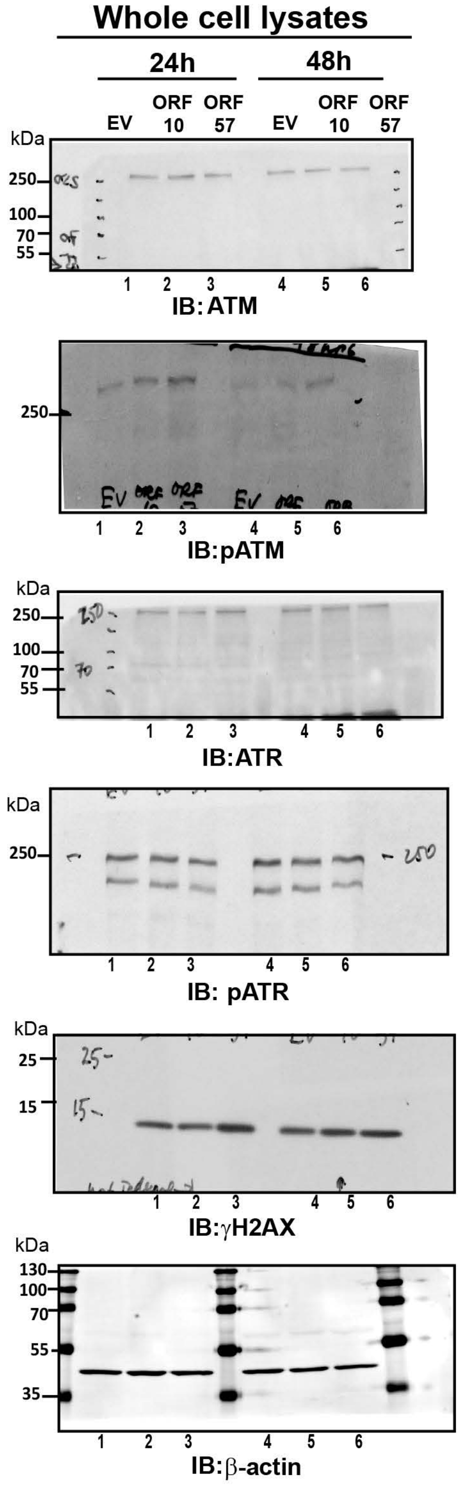

O Original images for Figure S4B

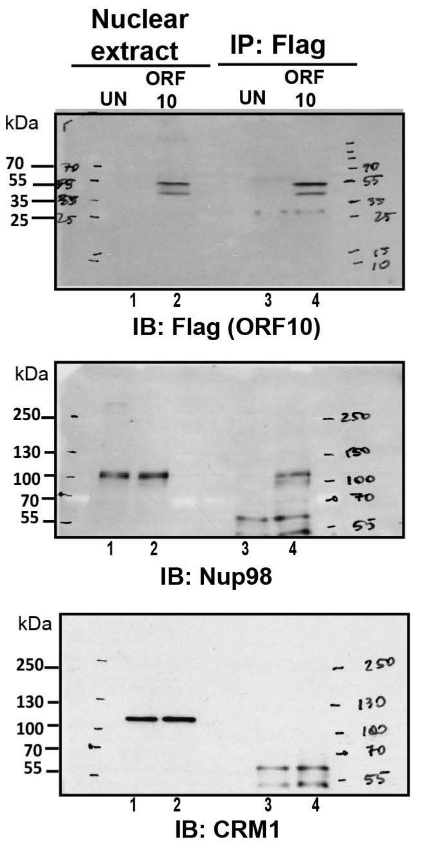

Supplement: S6 Fig — (PDF) [file ppat.1009033.s006.pdf]
