# Supplementary material for: Novel modulators of p53-signaling encoded by unknown genes of emerging viruses
Source: PLoS Pathog. 2021 Jan 7;17(1):e1009033. doi: 10.1371/journal.ppat.1009033 (PMC7790267; doi:10.1371/journal.ppat.1009033)
Supplement: S1 Table — Plasmid constructs expressing orfs encoded by ZIKV, CHIKV, EBOV, IFA, SARS, MERS-CoV, bat CoV, and KSHV were cloned using the gateway cloning system and were validated by sequencing, immunoblotting, and RT-PCR. Additional and updated information is available at the web-site that accompanies this manuscript: (https://www.med.unc.edu/orfeome). (PDF) [file ppat.1009033.s007.pdf]

Table S1.

Plasmid constructs expressing viral orfs screened in p53-Luc assays.

Plasmid constructs expressing orfs encoded by ZIKV, CHIKV, EBOV, IFA, SARS, MERS-CoV, bat CoV, and KSHV were cloned using the gateway cloning system and were validated by sequencing, immunoblotting, and RT-PCR. Additional and updated information is available at the web-site that accompanies this manuscript: (https://www.med.unc.edu/orfeome).

Construct	Vector	Virus	Strain (1)	ORF	Epitope
**pOME0001R**	pVR21	KSHV	JSC-1	K1	No
**pOME0002**	pDEST47	KSHV	JSC-1	K1	3xFlag
**pOME0002L**	pLENTI	KSHV	JSC-1	K1	3xFlag

**pOME0003R**	pVR21	KSHV	JSC-1	ORF10	No
**pOME0004**	pDEST47	KSHV	JSC-1	ORF10	3xFlag
**pOME0004L**	pLENTI	KSHV	JSC-1	ORF10	3xFlag
**pOME0005R**	pVR21	KSHV	JSC-1	K2	No
**pOME0006**	pDEST47	KSHV	JSC-1	K2	3xFlag
**pOME0006L**	pLENTI	KSHV	JSC-1	K2	3xFlag
**pOME0007R**	pVR21	KSHV	JSC-1	K3	No
**pOME0008**	pDEST47	KSHV	JSC-1	K3	3xFlag
**pOME0008L**	pLENTI	KSHV	JSC-1	K3	3xFlag
**pOME0009R**	pVR21	KSHV	JSC-1	K5	No
**pOME0010**	pDEST47	KSHV	JSC-1	K5	3xFlag
**pOME0012**	pDEST47	KSHV	JSC-1	K7	3xFlag
**pOME0013R**	pVR21	KSHV	JSC-1	ORF16	No
**pOME0014**	pDEST47	KSHV	JSC-1	ORF16	3xFlag
**pOME0015R**	pVR21	KSHV	JSC-1	ORF45	No
**pOME0016**	pDEST47	KSHV	JSC-1	ORF45	3xFlag
**pOME0016L**	pLENTI	KSHV	JSC-1	ORF45	3xFlag
**pOME0018**	pDEST47	KSHV	JSC-1	ORF47	3xFlag
**pOME0018R**	pVR21	KSHV	JSC-1	ORF47	3xFlag
**pOME0020**	pDEST47	KSHV	JSC-1	ORF53	3xFlag
**pOME0020R**	pVR21	KSHV	JSC-1	ORF53	3xFlag
**pOME0022**	pDEST47	Bat CoV	HKU3	NS3a ORF3	3xFlag
**pOME0022R**	pVR21	Bat CoV	HKU3	NS3a ORF3	3xFlag
**pOME0023R**	pVR21	Bat CoV	HKU3	NS3b ORF4a	No

**pOME0024**	pDEST47	Bat CoV	HKU3	NS3b ORF4a	3xFlag
**pOME0024L**	pLENTI	Bat CoV	HKU3	NS3b ORF4a	3xFlag
**pOME0026**	pDEST47	Bat CoV	HKU3	NS3c ORF4b	3xFlag
**pOME0026R**	pVR21	Bat CoV	HKU3	NS3c ORF4b	3xFlag
**pOME0027R**	pVR21	Bat CoV	HKU3	NS3D ORF5	No
**pOME0028**	pDEST47	Bat CoV	HKU3	NS3D ORF5	3xFlag
**pOME0028L**	pLENTI	Bat CoV	HKU3	NS3D ORF5	3xFlag
**pOME0032**	pDEST47	Bat CoV	HKU3	HKU3 strain, ORF4A	3xFlag
**pOME0036**	pDEST47	Bat CoV	HKU3	HKU3 strain, ORF5	3xFlag
**pOME0036R**	pVR21	Bat CoV	HKU3	HKU3 strain, ORF5	3xFlag
**pOME0040**	pDEST47	SARS CoV	Urbani	ORF3b nt25689-26153	3xFlag
**pOME0040Co**	pDEST47	SARS CoV	Urbani	ORF3b nt 25689-26153	3xFlag
**pOME0043R**	pVR21	SARS CoV	Urbani	ORF7a	No
**pOME0044**	pDEST47	SARS CoV	Urbani	ORF7a	3xFlag
**pOME0044L**	pLENTI	SARS CoV	Urbani	ORF7a nt 27273-27641	3xFlag
**pOME0045R**	pVR21	SARS CoV	Urbani	ORF7b	No
**pOME0046**	pDEST47	SARS CoV	Urbani	ORF7b	3xFlag
**pOME0048**	pDEST47	SARS CoV	Urbani	ORF8a	3xFlag
**pOME0050**	pDEST47	SARS CoV	Urbani	ORF8b	3xFlag
**pOME0051N**	pDEST47	SARS CoV	Urbani	ORF9b	No

**pOME0052**	pDEST47	SARS CoV	Urbani	ORF9b	3xFlag
**pOME0056**	pDEST47	Bat CoV	HKU3	ORF3b nt 25633-25762	3xFlag
**pOME0059R**	pVR21	Bat CoV	HKU3	ORF7a	No
**pOME0060**	pDEST47	Bat CoV	HKU3	ORF 7a	3xFlag
**pOME0060L**	pLENTI	Bat CoV	HKU3	ORF 7a nt 27217-27585	3xFlag
**pOME0062**	pDEST47	Bat CoV	HKU3	ORF7b	3xFlag
**pOME0062R**	pVR21	Bat CoV	HKU3	ORF7b	3xFlag
**pOME0063N**	pDEST47	Bat CoV	HKU3	ORF8a	No
**pOME0064**	pDEST47	Bat CoV	HKU3	ORF8a	3xFlag
**pOME0065N**	pDEST47	Bat CoV	HKU3	ORF9b	No
**pOME0066**	pDEST47	Bat CoV	HKU3	ORF9b	3xFlag
**pOME0078**	pDEST47	MERS CoV	EMC-1/2012	ORF 4a nt 25852-26181	3xFlag
**pOME0084**	pDEST47	MERS CoV	EMC-1/2012	ORF 8b nt 28762-29100	3xFlag
**pOME0086**	pDEST47	MERS CoV	England	ORF3 nt 25531-25842	3xFlag
**pOME0088**	pDEST47	MERS CoV	England	ORF4b	3xFlag
**pOME0088R**	pVR21	MERS CoV	England	ORF4b	3xFlag
**pOME0090**	pDEST47	MERS CoV	England	ORF5	3xFlag
**pOME0092**	pDEST47	MERS CoV	England	ORF8b mutant	3xFlag
**pOME0095**	pDEST47	EBOV	EBOV	NP Hypothetical gene #1	3xFlag
**pOME0097**	pDEST47	EBOV	EBOV	NP Hypothetical gene #3	3xFlag
**pOME0099**	pDEST47	EBOV	EBOV	GP Hypothetical gene #1	3xFlag

**pOME0099L**	pLENTI	EBOV	EBOV	GP Hypothetical gene #1	3xFlag
**pOME0100R**	pVR21	IFA	A/WSN/33	PA-X	No
**pOME0101**	pDEST47	IFA	A/WSN/33	PA-X	3xFlag
**pOME0102R**	pVR21	IFA	A/Brevig Mission 1/1918	M3	No
**pOME0103**	pDEST47	IFA	A/Brevig Mission 1/1918	M3	3xFlag
**pOME0103L**	pLENTI	IFA	A/Brevig Mission 1/1918	M3	3xFlag
**pOME0104**	pDEST47	EBOV	Lloviu	VP24	3xFlag
**pOME0105**	pDEST47	EBOV	Lloviu	VP35	3xFlag
**pOME0107**	pDEST47	EBOV	Lloviu	sGP	3xFlag
**pOME0116**	pDEST47	KSHV	JSC-1	ORF8	3xFlag
**pOME0128**	pDEST47	KSHV	JSC-1	ORF K4.1	3xFlag
**pOME0133**	pCDNA3.1 (+)	KSHV	JSC-1	downstream MIR-Syn	No
**pOME0133MR**	pSIREN	KSHV	JSC-1	downstream MIR-SYN	No
**pOME0134**	pCDNA3.1 (+)	KSHV	JSC-1	upstream MIR-SYN	No
**pOME0134MR**	pSIREN	KSHV	JSC-1	upstream MIR SYN	No
**pOME0135**	pCDNA3.1 (+)	KSHV	JSC-1	Kaposin cluster-RevCompl-SYN	No
**pOME0135MR**	pSIREN	KSHV	JSC-1	Kaposin cluster_RevComp1_SYN	No
**pOME0136**	pCDNA3.1 (+)	KSHV	JSC-1	Kaposin cluster RevCompl_KapATGdel_Syn	No

**pOME0136MR**	pSIREN	KSHV	JSC-1	Kaposin cluster RevComp1_KapATGdel_SYN	No
**pOME0140**	pDEST47	Bat CoV	HKU3	RBD 8810-9103	3xFlag
**pOME0142**	pDEST47	Bat CoV	HKU3	RBD nt 10049-10312	3xFlag
**pOME0142R**	pVR21	Bat CoV	HKU3	RBD nt 10049-10312	3xFlag
**pOME0146Co**	pDEST47	Bat CoV	HKU3	RBD nt 28614-28457	No
**pOME0148**	pDEST47	SARS CoV	MA	nt 734-1225	3xFlag
**pOME0148Co**	pDEST47	SARS CoV	MA	nt 734-1225	3xFlag
**pOME0150**	pDEST47	SARS CoV	MA	nt 2993-3295	3xFlag
**pOME0152Co**	pDEST47	SARS CoV	MA	nt 10067-10330	3xFlag
**pOME0154**	pDEST47	SARS CoV	MA	nt 20121-20709	3xFlag
**pOME0156Co**	pDEST47	SARS CoV	MA	nt 28583-28795	No
**pOME0158**	pDEST47	CHIKV	Sri Lankan	nsP1	3xFlag
**pOME0160**	pDEST47	CHIKV	Sri Lankan	nsP2	3xFlag
**pOME0162**	pDEST47	CHIKV	Sri Lankan	nsP3	3xFlag
**pOME0163R**	pVR21	CHIKV	Sri Lankan	Stress granule pos. contr. nsp4 SL	No
**pOME0164**	pDEST47	CHIKV	Sri Lankan	nsP4	3xFlag
**pOME0166**	pDEST47	CHIKV	Sri Lankan	Capsid	3xFlag
**pOME0169**	pDEST47	EBOV	Lloviu	VP24	No
**pOME0170**	pDEST47	EBOV	Lloviu	VP35	No
**pOME0174**	pDEST47	IFA	A/Anhui H7N9	NS Hypothetical gene #2	3xFlag
**pOME0176**	pDEST47	KSHV	JSC-1	ORF39 gM Hypothetical gene	3xFlag

**pOME0178**	pDEST47	KSHV	JSC-1	ORF22	3xFlag
**pOME0180**	pDEST47	KSHV	JSC-1	ORF50 Hypothetical gene	3xFlag
**pOME0182**	pDEST47	KSHV	JSC-1	K8.1 glycoprotein	3xFlag
**pOME0182R**	pVR21	KSHV	JSC-1	K8.1 Hypothetical gene	3xFlag
**pOME0184**	pDEST47	KSHV	JSC-1	ORF57	3xFlag
**pOME0186**	pDEST47	MERS CoV	EMC	nsp1	3xFlag
**pOME0188**	pDEST47	MERS CoV	EMC-1/2012	Envelope (E)	3xFlag
**pOME0188R**	pVR21	MERS CoV	EMC-1/2012	Envelope (E)	3xFlag
**pOME0190**	pDEST47	MERS CoV	EMC-1/2012	nsp1 altered	3xFlag
**pOME0192**	pDEST47	KSHV	JSC-1	ORF55	3xFlag
**pOME0204C**	pCAGGS	EBOV	EBOV	NP Hypothetical gene #2	3xFlag
**pOME0206**	pDEST47	EBOV	EBOV	Small secreted GP	3xFlag
**pOME0207MR**	pSIREN	KSHV	JSC-1	miR-K12-2wt	No
**pOME0208MR**	pSIREN	KSHV	JSC-1	miR-K12-4wt	No
**pOME0209MR**	pSIREN	KSHV	JSC-1	miR-K12-5wt	No
**pOME0210MR**	pSIREN	KSHV	JSC-1	miR-K12-6wt	No
**pOME0211MR**	pSIREN	KSHV	JSC-1	miR-K12-7wt	No
**pOME0212MR**	pSIREN	KSHV	JSC-1	miR-K12-9wt	No
**pOME0213MR**	pSIREN	KSHV	JSC-1	miR-K12-10wt	No
**pOME0215**	pDEST47	MERS CoV	England	ORF 8bwt nt 28729-29067	3xFlag
**pOME0219C**	pCAGGS	IFA	Ca04	PB1 Hypothetical gene #1	3xFlag
**pOME0223**	pDEST47	IFA	Ca04	PB2 Hypothetical gene #1	3xFlag

**pOME0231**	pDEST47	IFA	Mal/NY	NP Hypothetical gene #1	3xFlag
**pOME0232M**	pCDNA3.1 (-)	KSHV	JSC-1	Reverse compliment for KSHV downstream MIR-Syn	No
**pOME0233M**	pCDNA3.1 (-)	KSHV	JSC-1	Reverse compliment KSHV upstream MIR-SYN	No
**pOME0234M**	pCDNA3.1 (-)	KSHV	JSC-1	Reverse compliment of Kaposin cluster-RevCompl-SYN	No
**pOME0235M**	pCDNA3.1 (-)	KSHV	JSC-1	Reverse compliment for Kaposin cluster_RevCompl_KapATGdel_Syn	No
**pOME0236P**	pCDNA3.1 (+)	KSHV	JSC-1	anti-PAN	No
**pOME0237M**	pCDNA3.1 (-)	KSHV	JSC-1	Reverse compliment of anti-PAN	No
**pOME0238P**	pCDNA3.1 (+)	KSHV	JSC-1	antiRTA3kb	No
**pOME0239M**	pCDNA3.1 (-)	KSHV	JSC-1	Reverse compliment of antiRTA3kb	No
**pOME0240P**	pCDNA3.1 (+)	KSHV	JSC-1	T15	No
**pOME0241M**	pCDNA3.1 (-)	KSHV	JSC-1	Reverse compliment of T15	No
**pOME0242P**	pCDNA3.1 (+)	KSHV	JSC-1	antiRTA1kb	No
**pOME0243M**	pCDNA3.1 (-)	KSHV	JSC-1	reverse compliment antiRTA1kb	No
**pOME0244P**	pCDNA3.1 (+)	KSHV	JSC-1	PAN_RNA	No
**pOME0245M**	pCDNA3.1 (-)	KSHV	JSC-1	Reverse complimentl for PAN_RNA	No
**pOME0246P**	pCDNA3.1 (+)	KSHV	JSC-1	T61	No
**pOME0247M**	pCDNA3.1 (-)	KSHV	JSC-1	Reverse compliment of T61	No

**pOME0248MR**	pSIREN	EBOV	EBOV	GP-IGR VP30	No
**pOME0249MR**	pSIREN	EBOV	EBOV	VP 24 IGR L	No
**pOME0250MR**	pSIREN	EBOV	EBOV	VP35 IR VP40	No
**pOME0251R**	pVR21	CHIKV	Sri Lankan	5' UTR negative strand ORF	No
**pOME0252**	pDEST47	CHIKV	Sri Lankan	5' UTR negative strand ORF	3xFlag
**pOME0253MR**	pSIREN	SARS CoV	Urbani	3' UTR (29389-29727)	No
**pOME0254MR**	pSIREN	MERS CoV	EMC	3' UTR (29808-30107)	No
**pOME0255MR**	pSIREN	Bat CoV	HKU5	3' UTR (30162-30466)	No
**pOME0256MR**	pSIREN	Bat CoV	HKU4	3' UTR (29969-30270)	No
**pOME0258**	pDEST47	SARS CoV	MA	Nsp5	3xFlag
**pOME0260**	pDEST47	SARS CoV	MA	Nsp12	3xFlag
**pOME0262**	pDEST47	SARS CoV	MA	Nsp13	3xFlag

**pOME0264**	pDEST47	SARS CoV	MA	Nsp14	3xFlag
**pOME0268**	pDEST47	SARS CoV	MA	Envelope	3xFlag
**pOME0269R**	pVR21	CHIKV	Sri Lankan	Negative strand ORF 181/25	No
**pOME0270**	pDEST47	CHIKV	Sri Lankan	Negative strand ORF 181/25	3xFlag
**pOME0271P**	pCDNA3.1 (+)	KSHV	JSC-1	Alt lncRNA	No
**pOME0273P**	pCDNA3.1 (+)	KSHV	JSC-1	antiRTA3kb	No
**pOME0274M**	pCDNA3.1 (-)	KSHV	JSC-1	antiRTA3kb reverse compliment sequence control	No
**pOME0275P**	pCDNA3.1 (+)	KSHV	JSC-1	TO_7	No
**pOME0276M**	pCDNA3.1 (-)	KSHV	JSC-1	Reverse Compliment TO_7 control	No
**pOME0277P**	pCDNA3.1 (+)	KSHV	JSC-1	antiPAN	No
**pOME0278M**	pCDNA3.1 (-)	KSHV	JSC-1	antiPAN reverse compliment sequence control	No
**pOME0280**	pDEST47	EBOV	EBOV	VP40 HYPOTHETICAL GENE #1	3xFlag
**pOME0282**	pDEST47	EBOV	EBOV	NP HYPOTHETICAL GENE #5	3xFlag
**pOME0284**	pDEST47	IFA	A/WSN/33	PB2 S1	3xFlag
**pOME0286**	pDEST47	KSHV	JSC-1	ORF 36	3xFlag
**pOME0288**	pDEST47	KSHV	JSC-1	K15	3xFlag
**pOME0290M**	pCDNA3.1 (-)	KSHV	JSC-1	Downstream MIR-SYN_reverse	No
**pOME0291M**	pcDNA3.1 (-)	KSHV	JSC-1	Reverse Control for KSHV Upstream-Mir-Syn	No

**pOME0292M**	pCDNA3. 1 (-)	KSHV	JSC-1	Pan_RNA_Rev	No
**pOME0293M**	pcDNA3. 1 (-)	KSHV	JSC-1	anti-RTA_1Kb	No
**pOME0294M**	pCDNA3. 1 (-)	KSHV	JSC-1	anti-RTA_3Kb_Reverse	No
**pOME0295M**	pcDNA3. 1 (-)	KSHV	JSC-1	Kaposin cluster_RevCompl_Kap ATGdel_SYN_rev	No
**pOME0296M**	pcDNA3. 1 (-)	KSHV	JSC-1	Kaposin cluster_RevCompl_SYN _rev	No
**pOME0297M**	pcDNA3. 1 (-)	KSHV	JSC-1	T0_7-Rev	No
**pOME0298M**	pCDNA3. 1 (-)	KSHV	JSC-1	T15_reverse	No
**pOME0299M**	pCDNA3. 1 (-)	KSHV	JSC-1	T61_Reverse	No
**pOME0300M**	pDEST47	KSHV	JSC-1	antiPAN_Rev	No
**pOME0302**	pDEST47	ZIKV	H/PF/2013	NS1	3xFlag
**pOME0303R**	pVR21	ZIKV	H/PF/2013	NS2A	No
**pOME0304**	pDEST47	ZIKV	H/PF/2013	NS2A	3xFlag
**pOME0304L**	pLENTI	ZIKV	H/PF/2013	NS2A	No
**pOME0306**	pDEST47	ZIKV	H/PF/2013	NS2B	3xFlag
**pOME0308**	pDEST47	ZIKV	H/PF/2013	NS3	3xFlag
**pOME0310**	pDEST47	ZIKV	H/PF/2013	NS4A	3xFlag
**pOME0312**	pDEST47	ZIKV	H/PF/2013	PROTEIN 2K	3xFlag
**pOME0314**	pDEST47	ZIKV	H/PF/2013	NS4B	3xFlag
**pOME0316**	pDEST47	ZIKV	H/PF/2013	NS5	3xFlag
**pOME0318**	pDEST47	ZIKV	H/PF/2013	anchored capsid protein C	3xFlag

**pOME0320**	pDEST47	ZIKV	H/PF/2013	capsid protein C	3xFlag
**pOME0322**	pDEST47	ZIKV	H/PF/2013	membrane glycoprotein M	3xFlag
**pOME0326**	pDEST47	ZIKV	H/PF/2013	envelope protein E	3xFlag
